# *Caenorhabditis elegans* RIG-I-like receptor DRH-1 signals via CARDs to activate antiviral immunity in intestinal cells

**DOI:** 10.1073/pnas.2402126121

**Published:** 2024-07-09

**Authors:** Lakshmi E. Batachari, Alyssa Y. Dai, Emily R. Troemel

**Affiliations:** ^a^School of Biological Sciences, Department of Cell and Developmental Biology, University of California, San Diego, La Jolla, CA 92093

**Keywords:** RIG-I-like receptor, viral infection, innate immunity, *C. elegans*, intracellular pathogen response

## Abstract

Upon sensing viral RNA, mammalian RIG-I-like receptors (RLRs) activate downstream signals using caspase activation and recruitment domains (CARDs), which ultimately promote transcriptional immune responses that have been well studied. In contrast, the downstream signaling mechanisms for invertebrate RLRs are much less clear. For example, the *Caenorhabditis elegans* RLR DRH-1 lacks annotated CARDs and up-regulates the distinct output of RNA interference. Here, we found that similar to mammal RLRs, DRH-1 signals through two tandem CARDs (2CARD) to induce a transcriptional immune response. Expression of DRH-1(2CARD) alone in the intestine was sufficient to induce immune gene expression, increase viral resistance, and promote thermotolerance, a phenotype previously associated with immune activation in *C. elegans*. We also found that DRH-1 is required in the intestine to induce immune gene expression, and we demonstrate subcellular colocalization of DRH-1 puncta with double-stranded RNA inside the cytoplasm of intestinal cells upon viral infection. Altogether, our results reveal mechanistic and spatial insights into antiviral signaling in *C. elegans*, highlighting unexpected parallels in RLR signaling between *C. elegans* and mammals.

## Significance

Viruses are ubiquitous pathogens that challenge diverse organisms, from bacteria to killer whales. While antiviral defense has been well-studied in mammals, less is known about antiviral defense in invertebrates, including the roundworm *Caenorhabditis elegans*. Here, we show that *C. elegans* viral sensor dicer-related helicase 1 (DRH-1) shares similarities to a viral sensor in mammals called retinoic acid-inducible gene I (RIG-I). We find that DRH-1 has a tandem caspase activation and recruitment domain (2CARD) that promotes immunity and antiviral resistance, resembling 2CARD-mediated signaling in human RIG-I. Furthermore, we demonstrate that DRH-1, similar to RIG-I, forms clusters inside intestinal cells during viral infection. These findings provide insights into *C. elegans* antiviral immunity, highlighting parallels with mammalian antiviral immunity.

Over the last several years, the study of immune systems across diverse hosts has uncovered far broader evolutionary conservation in cytosolic innate immune responses than previously appreciated. In particular, recent findings highlight the striking conservation of antiviral immune pathways between bacteria and mammals (1). For example, the cyclic GMP-AMP synthase (cGAS)-stimulator of interferon genes (STING) pathway, which activates immune responses upon sensing cytosolic DNA in mammals, was recently identified in bacteria, where it detects bacteriophage infection and activates protective immune responses (2). In another example, cytosolic nucleotide-binding and leucine-rich repeat (NLR) receptors, which have long been known to be important in plant and mammalian immunity, have recently been identified in bacteria, where they also activate innate immune responses (3).

A central tenet of cell-intrinsic innate immunity is the recognition of pathogen- or microbe-associated molecular patterns (PAMPs or MAMPs) by germline-encoded receptors, called pattern recognition receptors (PRRs) (4), such as the cGAS and NLR receptors mentioned above. While great strides have been made in understanding the conservation in PRRs between mammalian and bacterial hosts, less is known about the PRRs of invertebrate hosts. In particular, we have a limited understanding of the specific PAMPs/MAMPs sensed by the nematode *Caenorhabditis elegans* and the PRRs that sense these PAMPs/MAMPs (5–8). In general, *C. elegans* lacks canonical PRRs, such as cGAS-STING and NLR receptors, which recognize broad classes of PAMPs/MAMPs. Arguably, the only known class of PRR that detects a general PAMP/MAMP and has been shown to be conserved between *C. elegans* and mammals is the RIG-I-like receptor (RLR) class. Mammalian RLRs, such as RIG-I and MDA5, sense cytosolic RNA to activate a type-I interferon (IFN-I) response, which is a transcriptional response critical for host defense against viral infection (9).

*C. elegans* has three RLRs—Dicer-related helicase DRH-1, -2, and -3—that share homology with mammalian RLRs at the helicase and C-terminal domains (CTD). Early work identified a role for DRH-1 in defense against RNA viruses through its role in directing RNA interference (RNAi) to degrade viral RNA (10–14). The viruses used in these studies included the Orsay virus, a single-stranded positive-sense RNA virus that is the only known natural viral pathogen of *C. elegans* (15). One of these early studies on antiviral RNAi demonstrated that the human RIG-I helicase and CTD could functionally substitute for the homologous domains in DRH-1, when measuring viral RNA levels (12). In mammals, the helicase domain and CTD bind dsRNA generated during RNA virus infection (16). Thus, the ability of human RIG-I helicase/CTD to function in place of DRH-1 helicase/CTD in *C. elegans* suggests that DRH-1 likely binds double-stranded RNA or other viral replication products. Consistent with this hypothesis, a recent cryo-EM analysis of DRH-1, along with its binding partners DCR-1 and RNA-binding protein RDE-4, indicates binding to blunt-ended double-stranded RNA (17).

**Fig. 1.**
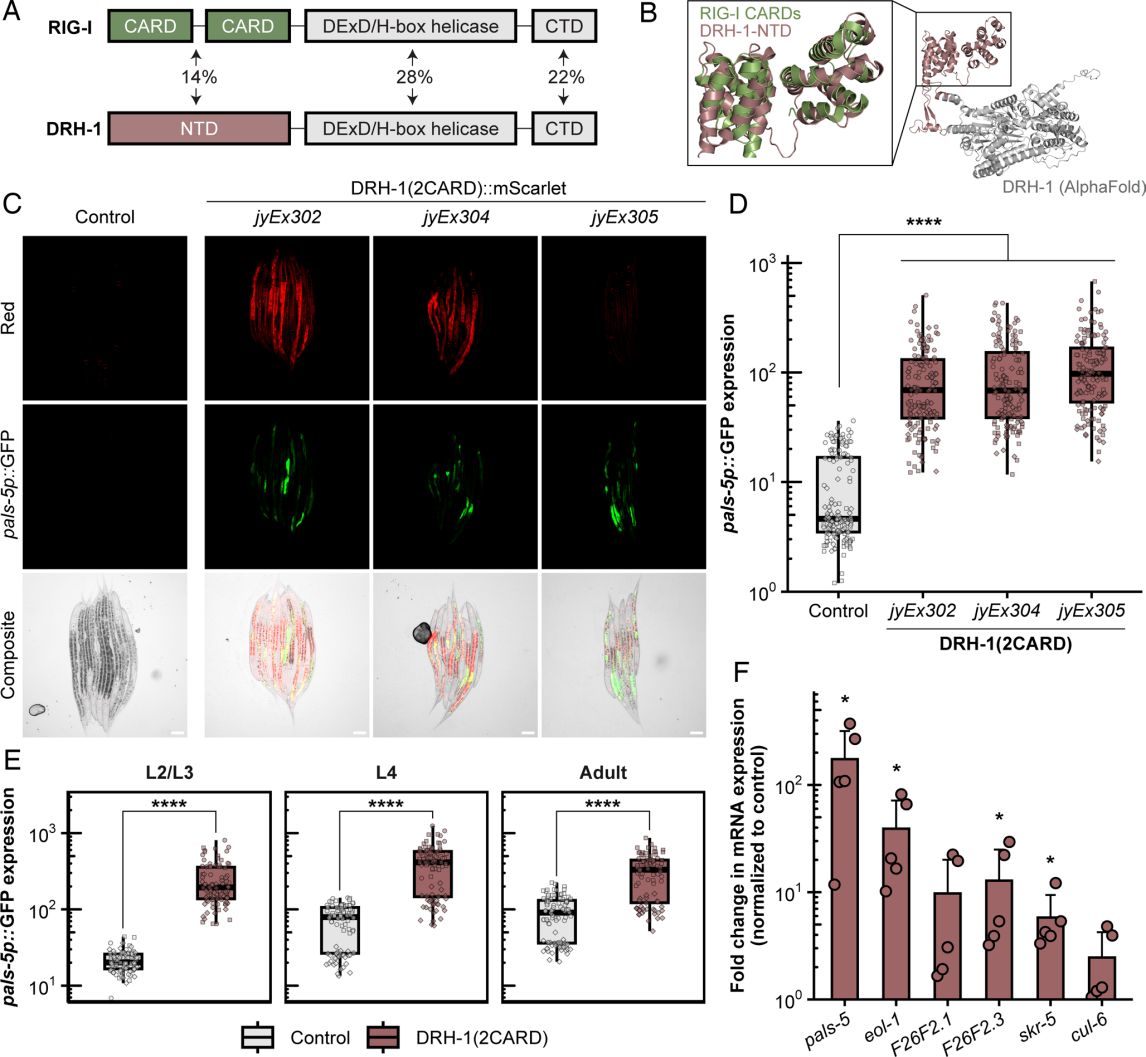
DRH-1 contains two predicted tandem CARDs that activate *pals-5p::*GFP and IPR gene expression. (*A*) Comparison of domain architecture and amino acid sequence similarity between RIG-I and DRH-1. Pairwise sequence alignment performed with Clustal Omega. (*B*) AlphaFold prediction of DRH-1 protein structure (white and dark salmon) with superimposition of RIG-I CARDs (green) at the N terminus (dark salmon). (*C*) Intestinal overexpression of DRH-1 NTD, or DRH-1(2CARD), induces *pals-5p::*GFP expression across three independent transgenic lines; *jyEx302, jyEx304*, and *jyEx305*. The designation “*jyEx”* indicates that the DRH-1(2CARD) transgene is expressed from an extrachromosomal array. The *myo-3p::*mCherry marker is expressed in body-wall muscle and is only present in line *jyEx302*, whereas *myo-2p::*mCherry is part of the *jyIs8[pals-5p::gfp]* transgene and is constitutively expressed in the pharynx in all three DRH-1(2CARD) lines and the control. (Scale bar, 50 µm.) (*D*) Quantification of *pals-5p*::GFP in the control strain and DRH-1(2CARD) transgenic lines shown in *C*. Each dot represents an individual animal; 150 animals were analyzed across three independent experiments for each strain. (*E*) DRH-1(2CARD) line *jyEx302* shows robust induction of *pals-5p::*GFP expression from larval stage L2 to adults. For each time point and genotype, 90 animals were analyzed across three independent experimental replicates. In both *D* and *E*, different dot symbols indicate different experimental replicates. Horizontal lines in box-and-whisker plots represent median values, and the box reflects the 25th to 75th percentiles. A Mann–Whitney *U* test was used to determine statistical significance in expression values between each transgenic line and the control; ****P < 0.0001. (*F*) DRH-1(2CARD) overexpression in line *jyEx305* (without the *pals-5p::*GFP reporter) up-regulates endogenous IPR gene expression. qRT-PCR analysis of a mixed-stage population containing both DRH-1(2CARD) transgenic animals and their nontransgenic siblings. Fold change in gene expression was determined relative to an *rde-1* mutant, nontransgenic control strain. Bars represent the mean across experimental replicates; error bars represent the SD. Each dot represents a biological replicate (a plate with a minimum of 2,000 animals); four independent experimental replicates were performed. A one-tailed *t* test was used to calculate *P*-values; *P < 0.05.

Relative to the helicase/CTD, the N-terminal domains (NTDs) of RIG-I and DRH-1 share less sequence similarity (Fig. 1*A*). The NTD of RIG-I contains two tandem caspase activation and recruitment domains (2CARD), which are homotypic interaction motifs. In RIG-I, the 2CARD binds the helicase/CTD to form an autoinhibited configuration in the absence of infection. Upon viral infection, the RIG-I helicase/CTD binds viral RNA replication products, which releases the N-terminal 2CARD to activate downstream signaling (16). In support of this model, the initial identification of RIG-I found that ectopic expression of RIG-I(2CARD) alone was sufficient to induce IFN-I gene expression (18). The RIG-I(2CARD) can directly interact with the CARD found in mitochondrial antiviral-signaling protein (MAVS), which promotes CARD oligomerization and downstream signal transduction to trigger the transcription of IFN-I genes. Because *C. elegans* lacks an obvious MAVS homolog and directs RNAi responses, the prevailing thought was that DRH-1 and mammalian RLRs had distinct signaling mechanisms during viral infection (19).

In prior work, we demonstrated that DRH-1 had a separate role from regulating RNAi. Namely, we found that DRH-1 was required for activating a transcriptional immune response in *C. elegans* termed the Intracellular Pathogen Response (IPR) (20). The IPR is triggered by infection with diverse intracellular pathogens that infect the intestine, including the Orsay virus and obligate intracellular fungi called microsporidia (21). While work from other groups indicated that DRH-1 directs an antiviral RNAi response through interactions with RNAi components, such as DCR-1 and RDE-4, and downstream signaling components RDE-1 and DRH-3 (10, 11, 13), we found that these RNAi factors are dispensable for IPR activation during viral infection (20). However, we did find that heterologous expression of a replication-competent Orsay virus RNA1 genome segment induced most of the IPR genes, dependent on DRH-1, again indicating that DRH-1 senses dsRNA or some other viral replication product (20, 22). Downstream of DRH-1, we have found that the bZIP transcription factor ZIP-1 activates a subset of the IPR genes (23), but otherwise, it remains unclear how DRH-1 signals to induce the IPR upon viral infection.

In this study, we show that intestine-specific expression of the DRH-1 NTD alone is sufficient to induce the IPR in a partially ZIP-1-dependent manner. Despite the low primary sequence similarity between DRH-1 NTD and RIG-I(2CARD), we find that AlphaFold (24, 25) predictions indicate high three-dimensional similarity between these two proteins. Ectopic expression of DRH-1(2CARD) (aka DRH-1 NTD) in the intestine increases resistance to viral infection and promotes thermotolerance. Furthermore, we show that DRH-1 is required in the intestine for response to infection. Our subcellular analyses indicate that full-length DRH-1 protein forms puncta inside intestinal cells upon infection, and these puncta colocalize with viral and double-stranded RNA. Overall, these findings advance our understanding of how DRH-1 signals to activate a transcriptional immune response, revealing surprising similarities between mammalian RLRs and *C. elegans* DRH-1.

## Results

### The N terminus of DRH-1 Has Two Predicted Tandem CARDs (2CARD) that Activate IPR Gene Expression.

The NTD of mammalian RIG-I contains 2CARDs, also known as 2CARD, which are each composed of six alpha-helices (Fig. 1*B*). Mammalian CARDs have been extensively studied for their role in innate immune signaling pathways. In particular, CARDs mediate signal transduction through interactions with other CARDs. An example of CARD-CARD-mediated antiviral signaling involves the interaction of RIG-I CARDs and the CARD found in downstream signaling protein MAVS (16). To investigate structural similarity in the NTDs of DRH-1 and RIG-I, we superimposed the CARDs of human RIG-I [Protein Data Bank (PDB) ID: 4p4h] onto the AlphaFold prediction of DRH-1 NTD. The two structures exhibited extensive three-dimensional similarity, including a pair of six antiparallel alpha-helices that overlaid well between the N-terminal 2CARD of RIG-I and predicted structure for DRH-1 NTD (Fig. 1*B*). When comparing RIG-I to DRH-1, CARD1 and CARD2 have rmsd values of 5.6 Å and 5.1 Å, respectively (0 Å is a perfect match). Of note, a comparison of RIG-I CARD1 to CARD2 gives an rmsd value of 5.1 Å, indicating the level of divergence that can be found between two different sequences both annotated as CARDs. Thus, the predicted structure for the NTD of DRH-1 resembles the 2CARDs found in the NTD of mammalian RLRs.

In an untargeted approach to identify proteins with structural similarity to the DRH-1 NTD, we used Foldseek (26) to identify matches between the predicted structure of DRH-1 NTD and known structures deposited in the PDB. The top six structural matches corresponded to CARDs, with human and mouse 2CARDs at the top of the list (Dataset S1). Next, we used the Dali structure alignment tool. Similar to the results from Foldseek, we found with Dali analysis that human RIG-I(2CARD) was the top structural match to DRH-1 NTD (Dataset S2). Recent structural analysis has revealed that DRH-3, a distinct *C. elegans* RLR with a known role in RNAi (10, 11, 13) but so far not a demonstrated role in transcriptional responses (20), also contains two N-terminal tandem CARDs (27). Notably, the structure for DRH-3 CARDs (PDB ID: 6m6q) was listed as the 7th top structural match for DRH-1 NTD based on the comparisons performed with Dali (Dataset S2). This finding lends additional support to the hypothesis that the NTD of DRH-1 contains 2CARD. For these reasons, as well as the RMSD values discussed above, we here-on refer to DRH-1 NTD as DRH-1(2CARD).

Though *C. elegans* lacks obvious IFN and IFN receptor homologs, a collection of findings indicates parallels in the regulation of the IPR in *C. elegans* and the regulation of IFN-I signaling in mammals (9). Given that ectopic expression of RIG-I(2CARD) is sufficient to induce IFN-I signaling in mammals (18), we explored whether DRH-1(2CARD) expression would be sufficient to induce IPR signaling in *C. elegans*. As Orsay virus infects intestinal cells, we used an intestine-specific promoter, *vha-6p*, to drive overexpression of mScarlet-tagged DRH-1(2CARD). Here, we found that animals expressing DRH-1(2CARD) exhibited induction of the *pals-5p::*GFP reporter, a commonly used read-out for IPR induction. This effect was seen across three independent extrachromosomal transgenic lines (Fig. 1 *C* and *D*), and was not seen in nontransgenic siblings (*SI Appendix*, Fig. S1 *A* and *B*). This effect was also consistent across developmental stages from the second larval stage through adult (Fig. 1*E*). As a negative control, we created transgenic animals carrying a construct that lacks DRH-1(2CARD), but contains all other components of the vector, including *vha-6p* and mScarlet. *pals-5p::*GFP expression was not induced in that negative control strain (*SI Appendix*, Fig. S1*C*). We also demonstrated that there is no *pals-5p::*GFP induction in animals with intestine-specific expression of the DRH-1 helicase/CTD, referred to as DRH-1(HC), tagged with mScarlet (*SI Appendix*, Fig. S1*D*), again demonstrating that *pals-5p::*GFP induction is specific to expression of DRH-1(2CARD).

To examine whether DRH-1(2CARD) might induce other IPR genes, we analyzed its effects on a GFP reporter for the expression of *F26F2.1*, another highly induced IPR gene of unknown function (28). Here as well, we saw significant induction of *F26F1.1p*::GFP by DRH-1(2CARD) expression (*SI Appendix*, Fig. S2 *A* and *B*). To confirm that these IPR reporters reflected endogenous gene expression, we performed qRT-PCR analysis and found that DRH-1(2CARD) significantly up-regulated expression of endogenous mRNA for *pals-5*, *F26F2.1*, as well as other IPR genes (Fig. 1*F* and *SI Appendix*, Fig. S2*C*).

**Fig. 2.**
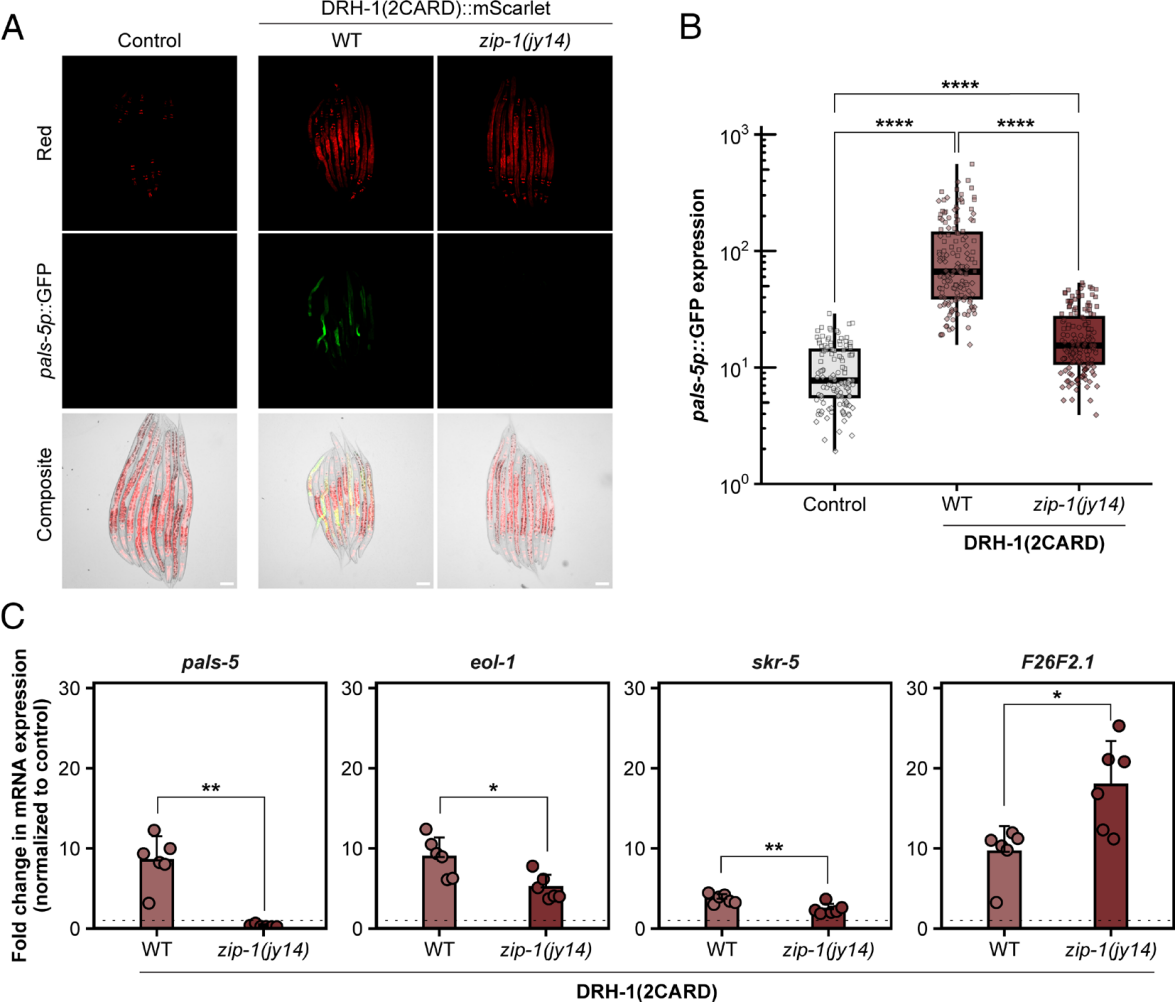
DRH-1(2CARD) overexpression up-regulates *pals-5p::*GFP and *pals-5* mRNA in a pathway that involves the transcription factor *zip-1.* (*A*) DRH-1(2CARD) overexpression in line *jyEx305* activates *pals-5p::*GFP in a manner that is partially dependent on the transcription factor *zip-1. myo-2p::*mCherry is part of the *jyIs8[pals-5p::gfp]* transgene and is constitutively expressed in the pharynx of DRH-1(2CARD) transgenic lines, as well as in a control strain (*Left*) that does express the DRH-1(2CARD) transgene. (Scale bar, 50 µm.) (*B*) Quantification of *pals-5p*::GFP in strains shown in panel *A*. Different dot symbols indicate different experimental replicates. Horizontal lines in box-and-whisker plots represent median values, and the box reflects the 25th to 75th percentiles. A Kruskal–Wallis test with Dunn’s multiple comparisons test was used to calculate *P*-values; ****P < 0.0001. (*C*) Transcriptional upregulation of endogenous *pals-5, eol-1*, and *skr-5* expression by DRH-1(2CARD) is dependent on *zip-1*, whereas upregulation of *F26F2.1* expression is independent of *zip-1.* qRT-PCR analysis was performed on a mixed population of DRH-1(2CARD) animals in a WT background or *zip-1* mutant background. Bars represent the mean across experimental replicates; error bars represent the SD. Each dot represents a biological replicate (a plate with a minimum of 2,000 animals); three independent experimental replicates were performed. A *t* test was used to calculate *P*-values; *P < 0.05, **P < 0.01.

The IPR includes hundreds of genes highly up-regulated by viral infection, and about one-third of the top 80 genes depend on the bZIP transcription factor ZIP-1 for their expression, including *pals-5* (23, 28–30). To determine whether ZIP-1 was required for IPR induction by DRH-1(2CARD), we analyzed *pals-5p::*GFP in *zip-1* null mutants expressing the DRH-1 (2CARD) transgene. Here, we found significantly reduced *pals-5p::*GFP expression in the *zip-1* mutant background compared to the wild-type background (Fig. 2 *A* and *B*). We also used qRT-PCR to demonstrate that DRH-1(2CARD) induced expression of endogenous *pals-5* mRNA in a *zip-1*-dependent manner (Fig. 2*C*). Furthermore, we found significantly reduced expression of *eol-1* and *skr-5* mRNA in *zip-1* mutants, as well as significantly increased expression of *F26F2.1* in *zip-1* mutants, consistent with previous findings about ZIP-1-dependent and ZIP-1-independent IPR genes. Altogether these findings indicate that DRH-1(2CARD) induces IPR gene expression in a manner similar to other IPR triggers, like viral infection and the proteasome inhibitor bortezomib.

ZIP-1::GFP localizes to the nucleus in response to known IPR triggers, such as Orsay virus infection and the proteasome blocker bortezomib (*SI Appendix*, Fig. S3 *A* and *B*) (23). However, DRH-1(2CARD) expression was not sufficient to cause obvious nuclear localization of ZIP-1::GFP (*SI Appendix*, Fig. S3 *A* and *B*). Of note, prior studies have demonstrated that *zip-1* is required to induce early IPR gene expression, and only after prolonged IPR activation does ZIP-1::GFP become visible in the nucleus by fluorescence microscopy (23). Thus, it may be that in the data shown in Fig. 2, DRH-1(2CARD) expression is not a potent enough trigger (compared to prolonged infection and proteasome blockade), or needs an additional trigger, to promote visible levels of nuclear ZIP-1::GFP. Regardless, our genetic results indicate that *zip-1* plays a significant role in mediating induction of *pals-5* and other IPR genes upon expression of DRH-1(2CARD) (Fig. 2).

### DRH-1(2CARD) Expression in the Intestine Induces Several IPR Phenotypes, Including Resistance to Viral Infection and Heat Shock.

**Fig. 3.**
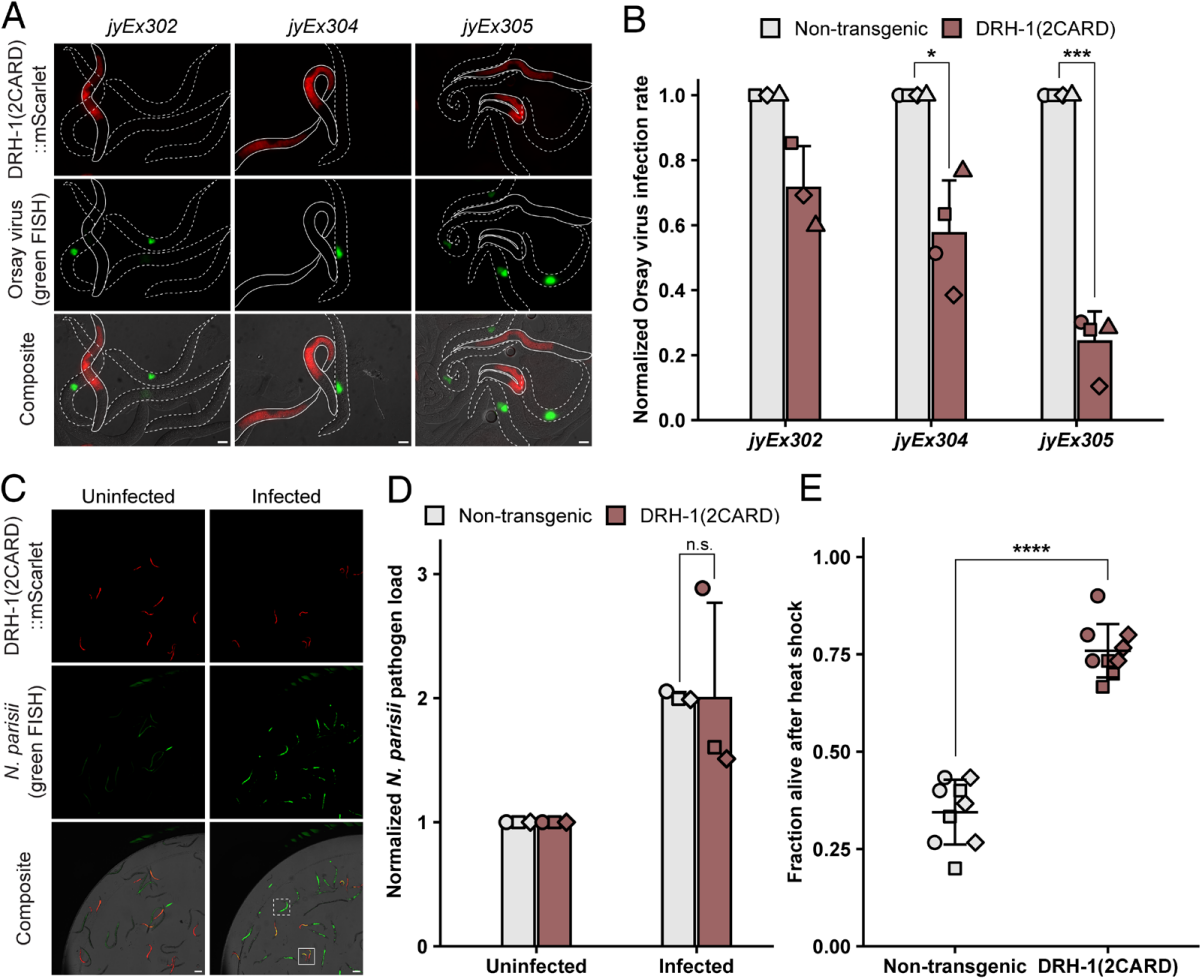
IPR activation by DRH-1(2CARD) reduces viral infection rate and increases thermotolerance but does not decrease *N. parisii* pathogen load. (*A*) Representative images of DRH-1(2CARD) transgenic animals (solid white outline) and nontransgenic siblings (dotted white outline) infected with Orsay virus. Viral infection was visualized using fluorescein-conjugated (green) FISH probes targeting the Orsay virus genome. Virus-infected cells are shown in green. (Scale bar, 20 µm.) (*B*) DRH-1(2CARD) confers increased resistance to viral infection at 18 hours post inoculation (hpi) relative to nontransgenic siblings across all three transgenic lines. Within each transgenic line, the fraction of the population infected with virus was determined by scoring animals based on the presence of green fluorescence. Normalized infection rate was determined by setting the infection rate of nontransgenic siblings to 1. For transgenic lines *jyEx305* and *jyEx304*, 400 animals were scored across n = 4 independent experimental replicates. For line *jyEx302*, 300 animals were scored across n = 3 independent experimental replicates. A *t* test was used to calculate *P*-values; ***P < 0.001; *P < 0.05. (*C*) Representative images of DRH-1(2CARD) transgenic animals and nontransgenic siblings infected with *N. parisii.* Infection was visualized by staining with green FISH probes targeting *N. parisii* ribosomal RNA. Green fluorescence in uninfected animals is due to autofluorescence. White boxes indicate the presence of *N. parisii* FISH fluorescent signal in DRH-1(2CARD) animals (solid border) and nontransgenic siblings (dashed border). (Scale bar, 100 µm.) (*D*) DRH-1(2CARD) expression does not reduce *N. parisii* pathogen load at 30 hpi. Normalized *N. parisii* infection rate was calculated by setting normalized green fluorescence values in uninfected worms to 1. Each dot represents an experimental replicate; three independent experimental replicates were performed. Uninfected: n = 1,011 (nontransgenic) or 1,435 [DRH-1(2CARD)]. Infected: n = 925 (nontransgenic) or 1,292 [DRH-1(2CARD)]. A Mann–Whitney *U* test was used to determine significance; n.s. = not significant. (*E*) DRH-1(2CARD) animals exhibit increased thermotolerance relative to nontransgenic siblings. Survival was scored after a 2 h heat shock at 37.5 °C followed by a 24 h incubation at 20 °C. Nine biological replicates (n = 9 plates) were scored over three independent experimental replicates. Thirty animals were scored per plate. A two-tailed *t* test was used to calculate *P*-values; ****P < 0.0001. Mean values are represented by bar height (*B* and *D*) or cross bar (*E*); error bars represent the SD. Different dot symbols indicate different experimental replicates.

To determine whether the IPR gene induction caused by DRH-1(2CARD) leads to increased resistance to viral infection, we measured the infection rate in a population containing both DRH-1(2CARD) extrachromosomal transgenic animals and their nontransgenic siblings. Across three transgenic lines, animals expressing DRH-1(2CARD) exhibited a decreased infection rate relative to their nontransgenic siblings (Fig. 3 *A* and *B* and *SI Appendix*, Fig. S3*A*). In contrast to the viral infection rate phenotype, we did not observe significantly increased resistance to the microsporidian intracellular pathogen *Nematocida parisii* upon quantifying pathogen load (Fig. 3 *C* and *D*). A priori, we would have expected to see increased resistance to *N. parisii*, as constitutive expression of IPR genes in other genetic backgrounds leads to increased resistance against both Orsay virus and *N. parisii* (30–33). A potential explanation for the lack of resistance to *N. parisii* may be that the DRH-1(2CARD) induces a subset of genes important for viral resistance but not *N. parisii* resistance. To investigate this hypothesis, we identified genes that might be more strongly induced upon *N. parisii* infection compared to virus infection, based on a published RNAseq dataset (34). We measured the expression of five of these genes, which include the IPR genes *math-38* and *clec-60*, to determine whether DRH-1(2CARD)-expressing animals lacked induction of these genes. However, we found all five of them were induced upon DRH-1(2CARD) overexpression (*SI Appendix*, Fig. S4 *B* and *C*), suggesting that differences in the expression of these infection-related genes are not responsible for the lack of resistance to *N. parisii*. Overall, our findings indicate that while DRH-1(2CARD) did not provide protection against *N. parisii*, it did provide robust protection against viral infection (Fig. 3*B*).

In addition to pathogen resistance, activation of the IPR is associated with several other phenotypes, including slowed development and increased resistance to heat shock (29, 35). Indeed, we observed impaired development in DRH-1(2CARD) transgenic animals compared to their nontransgenic siblings (*SI Appendix*, Fig. S4 *D* and *E*). Furthermore, we found a substantial increase in thermotolerance or resistance to heat shock. Specifically, ~75% of DRH-1(2CARD) transgenic animals survived 24 h (h) after a 2 h 37.5 °C heat shock, compared to only ~35% survival of their nontransgenic siblings (Fig. 3*E*). Taken together, these observations are consistent with the model that DRH-1(2CARD) expression activates the IPR, resulting in developmental and heat shock phenotypes similar to what has been previously observed in the context of other IPR triggers.

Next, we explored whether intestinal overexpression of full-length DRH-1 would promote the same phenotypes as overexpression of DRH-1(2CARD). However, in our attempts to generate transgenic lines carrying *vha-6p::drh-1::mScarlet*, we found that overexpression of full-length DRH-1 led to complete larval arrest, despite multiple attempts to generate transgenic animals (*SI Appendix*, Fig. S4*F*). Interestingly, we found that 100% of these transgenic progeny (n = 103 transgenic progeny across four injections) exhibited *pals-5p::*GFP activation, indicative of IPR activation. Therefore, while the intestine-specific full-length DRH-1 appears to activate the IPR, the phenotype of complete larval arrest prevented our ability to assess other IPR phenotypes. Of note, we have previously observed complete larval arrest in genetic backgrounds that cause very strong IPR activation, such as null mutants of *pals-17*, a gene encoding a negative regulator of the IPR that is expressed predominantly in the intestine (33).

### DRH-1 Is Required in the Intestine for IPR Induction and Forms Puncta upon Infection that Colocalize with Viral RNA.

**Fig. 4.**
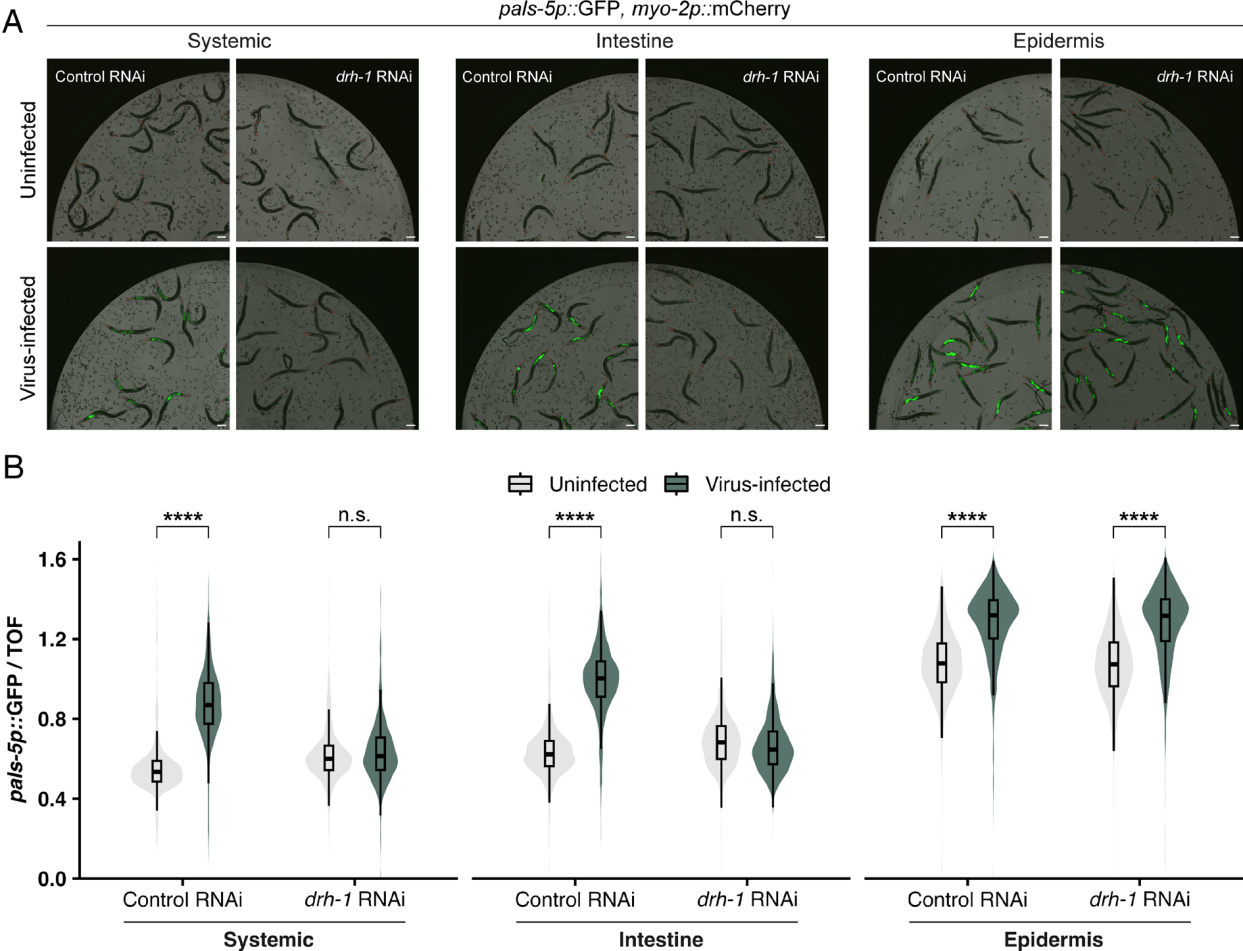
DRH-1 is required in the intestine to up-regulate *pals-5p::*GFP expression during viral infection. (*A*) Representative images of *pals-5p*::GFP expression upon systemic, intestine-specific, or epidermis-specific knockdown of *drh-1* during viral infection. *myo-2p::*mCherry is a part of the *jyIs8[pals-5p::gfp]* transgene and is constitutively expressed in the pharynx. Animals were treated with an empty vector control RNAi (L4440) or *drh-1* RNAi and infected with virus at the L4 stage for 24 h. (Scale bar, 100 µm.) See *SI Appendix*, Fig. S5 for quantification of *drh-1* knock-down. (*B*) Quantification of *pals-5p::*GFP fluorescence shown in *A* using a COPAS Biosort instrument. Knockdown of *drh-1* in the intestine, but not the epidermis, blocks the induction of *pals-5p::*GFP upon viral infection. *pals-5p*::GFP fluorescence is normalized to time of flight (a measure of worm size). The epidermis-specific RNAi strain has overall higher levels of *pals-5p::*GFP compared to the other strains. This difference is likely is due to the absence of functional *rde-1* in intestinal cells in this strain, resulting in impaired transgene silencing and increased baseline expression of *pals-5p*::GFP. Dots represent individual animals. Systemic: n = 961 animals (control RNAi; uninfected), 741 (control RNAi; infected), 1,035 (*drh-1* RNAi; uninfected), or 841 (*drh-1* RNAi; uninfected). Intestine: n = 779 (control RNAi; uninfected), 784 (control RNAi; infected), 951 (*drh-1* RNAi; uninfected), or 924 (*drh-1* RNAi; uninfected). Epidermis: n = 1,474 (control RNAi; uninfected), 1,564 (control RNAi; infected), 1,961 (*drh-1* RNAi; uninfected), or 1,655 (*drh-1* RNAi; uninfected). Horizontal lines in box-and-whisker plots represent median values, and the box reflects the 25th to 75th percentiles. For each tissue-specific knockdown strain, a Mann–Whitney *U* test was used to determine significant differences between uninfected vs. infected worms after treatment with control or *drh-1* RNAi; ****P < 0.0001.

The results above indicated that ectopic expression of DRH-1 alone in the intestine was sufficient to induce the IPR. scRNAseq analysis suggests that *drh-1* is expressed in intestinal tissue (36), and our prior work has shown that IPR activation can occur in either the intestine or the epidermis (29, 32). Therefore, we investigated whether DRH-1 is required in either of these tissues for IPR induction by implementing an experimental system that enables gene silencing in a tissue-specific manner. In particular, we used *C. elegans rde-1(ne300)* mutant strains where a wild-type copy of the RNAi factor *rde-1* is expressed only in the intestine or only in the epidermis, in order to rescue RNAi competency in these tissues (37). [Of note, *rde-1(ne300)* null mutants provide more tissue-specificity compared to the widely used partial loss-of-function *rde-1(ne219)* mutants, which are not fully RNAi-deficient.] Here, we found that, similar to systemic RNAi against *drh-1*, intestine-specific knockdown of *drh-1* blocked *pals-5p::*GFP induction upon viral infection (Fig. 4 and *SI Appendix*, Fig. S5). However, epidermis-specific knockdown of *drh-1* did not block *pals-5p::*GFP induction upon viral infection. Therefore, *drh-1* appears to be required in the intestine to induce the IPR upon viral infection.

To further understand how DRH-1 signals in intestinal cells, we investigated the subcellular localization of DRH-1 upon viral infection. Here, we first investigated full-length DRH-1, and because intestine-specific expression of full-length DRH-1 caused larval arrest (*SI Appendix*, Fig. S3*D*), we turned to a previously generated strain containing a single-copy insertion of the *mScarlet::drh-1* transgene driven by the ubiquitous promoter *rpl-28p* (38). This strain exhibited normal development, possibly due to lower *drh-1* expression levels from a single-copy transgene, compared to the multicopy, extrachromosomal transgene that caused arrest (*SI Appendix*, Fig. S3*D*). Here, we found that expression of *rpl-28p::mScarlet::drh-1* did not lead to ectopic expression of IPR genes in the absence of infection (*SI Appendix*, Fig. S6*A*). By crossing the *rpl-28p::mScarlet::drh-1* transgene into a *drh-1* null mutant background, we found that it rescued *drh-1* mutant phenotypes. In particular, we used qRT-PCR to show that *rpl-28p::mScarlet::drh-1* rescued mRNA expression of endogenous IPR genes during viral infection (*SI Appendix*, Fig. S6*A*). Transgene expression also reduced the increased viral load phenotype of *drh-1* mutants (*SI Appendix*, Fig. S6*B*). These results indicate that the *rpl-28p::mScarlet::drh-1* transgene in this strain is functional, so we used this strain to further analyze DRH-1 protein localization inside intestinal cells.

**Fig. 5.**
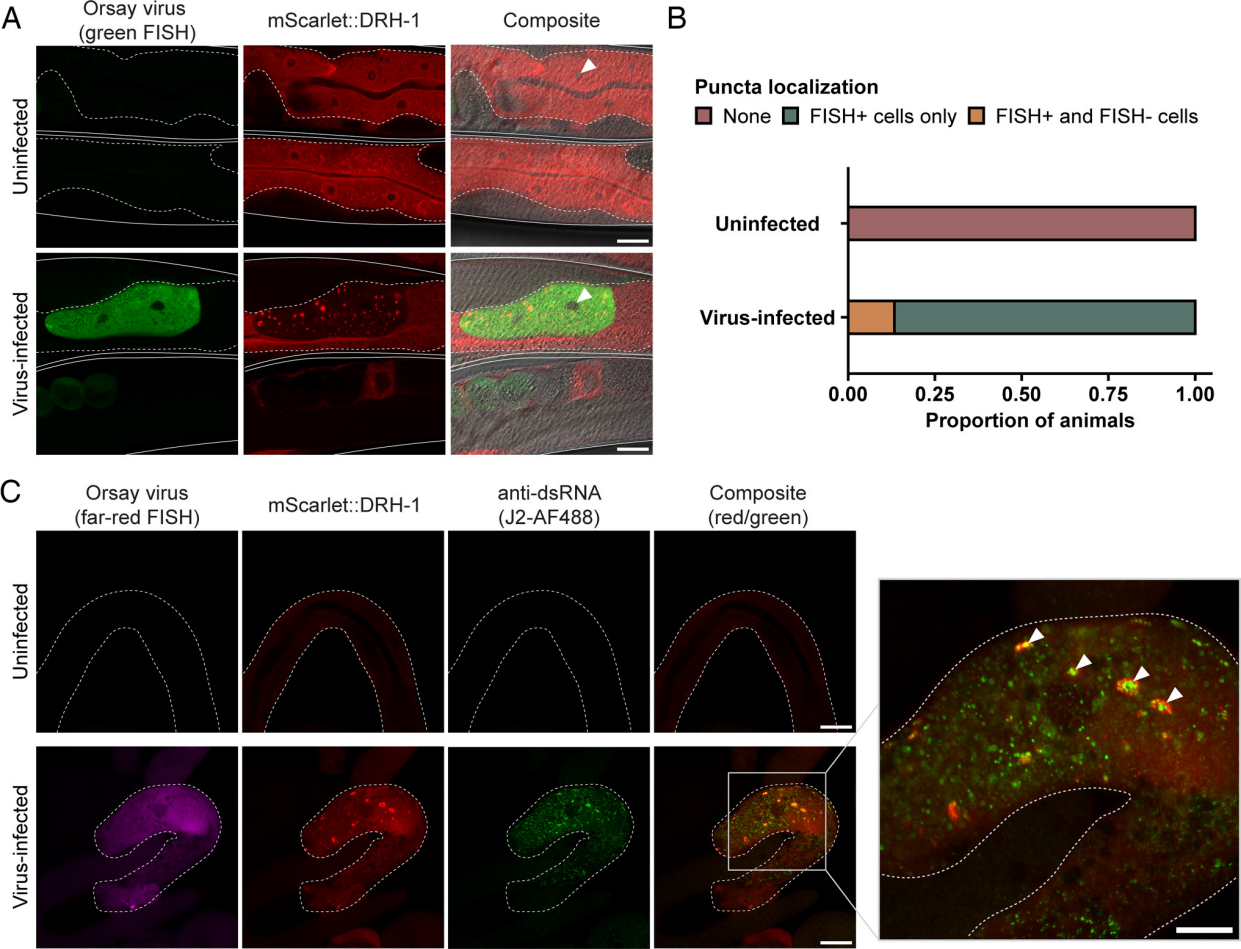
Virus-infected animals exhibit DRH-1 puncta in intestinal cells that colocalize with dsRNA. (*A*) Representative images of virus-infected adult animals that express mScarlet-tagged DRH-1 (red) as an integrated, single-copy transgene. Viral infection was visualized using fluorescein-conjugated (green) FISH probes targeting the Orsay virus genome. mScarlet::DRH-1 forms puncta (red) in virus-infected cells (green). Individual worms are outlined by solid white lines, and intestines are outlined by dotted white lines. White arrowheads indicate cell nuclei. (Scale bar, 25 µm.) (*B*) Quantification of puncta localization in uninfected animals or animals exposed to virus. DRH-1 puncta are only present in virus-infected animals. For each treatment condition, 30 animals were scored across at least three independent experimental replicates. (*C*) Representative images of dissected intestines (white dotted outline) from uninfected or virus-infected mScarlet::DRH-1 adult animals. Viral infection was visualized using Quasar 670-conjugated (far-red) FISH probes targeting the Orsay virus genome. The J2 antibody was used to visualize dsRNA. White arrowheads indicate colocalization between mScarlet::DRH-1 (red) and dsRNA (green) in virus-infected intestinal cells (magenta). Five worms were scored for each treatment condition. (Scale bar, 25 µm.)

First, we infected mScarlet::DRH-1 transgenic animals with Orsay virus and visualized virus-infected cells by fluorescence in situ hybridization (FISH) after 24 h of infection. Uninfected control animals exhibited a homogenous distribution of mScarlet::DRH-1 throughout the intestinal cell cytoplasm (Fig. 5*A*). In contrast, upon infection, mScarlet::DRH-1 formed discrete puncta in virus-infected intestinal cells (Fig. 5*A*). When we quantified this effect, we found that 0% of uninfected animals exhibited DRH-1 puncta, while 100% of infected animals exhibited DRH-1 puncta (Fig. 5*B*). In these experiments, animals often had a mix of infected and uninfected intestinal cells, and in some of these cases we found DRH-1 puncta in uninfected intestinal cells. This result suggests that there may be signaling from uninfected to infected intestinal cells to activate DRH-1 signaling and puncta formation (Fig. 5*B*), although an alternative possibility is that the Orsay virus FISH signal was below the level of detection in the cells we scored as uninfected.

To investigate which domain of DRH-1 is responsible for forming puncta upon viral infection, we used our DRH-1(2CARD) and DRH-1(HC)-expressing strains and analyzed subcellular localization. Here, we observed the formation of both puncta (localized fluorescence with an area less than 40 µm^2^) and aggregates (fluorescent area greater than 40 µm^2^) in DRH-1(2CARD) animals in the absence of infection (*SI Appendix*, Fig. S7*A*). To calculate puncta/aggregate density, the number of puncta/aggregates was normalized to the area of the intestinal region that was analyzed. Notably, DRH-1(2CARD) puncta and aggregate density were both significantly decreased upon viral infection in FISH-positive cells, with the median aggregate density decreasing to 0 (*SI Appendix*, Fig. S7 *B* and *C*). In contrast, DRH-1(HC) animals exhibited minimal puncta and aggregate formation in the absence of infection (*SI Appendix*, Fig. S7 *D*–*F*). Specifically, our analysis demonstrated that DRH-1(HC) animals had a median puncta density of 0 in uninfected cells, whereas FISH-positive cells from infected animals showed a significant increase in the median puncta density (3,906 puncta/mm^2^) relative to the uninfected control. The size of DRH-1(HC) puncta also appears larger in FISH-positive cells, resembling observations made with full-length DRH-1 puncta (Fig. 5). Altogether, these results indicate that the helicase and C-terminal domain of DRH-1 mediate puncta formation upon viral infection.

Next, we analyzed colocalization of full-length DRH-1 with viral replication products, using an antibody that recognizes dsRNA, in order to assess whether DRH-1 may colocalize with this viral replication product. Indeed, we found that in contrast to uninfected animals, infected animals exhibited extensive dsRNA antibody staining, which often colocalized with DRH-1 puncta (Fig. 5*C*). These findings are intriguing in light of studies in mammalian cells, where RIG-I forms puncta in response to viral infection and in the presence of viral RNA (39). RLR signal transduction occurs through CARD-carrying downstream signaling factor MAVS, which also forms puncta inside cells upon infection (40, 41). These mammalian studies in cell culture, paired with oligomerization studies in vitro (42, 43), highlight similarities to our findings here that *C. elegans* DRH-1 forms puncta that colocalize with viral replication products in the intestinal cells of an intact animal (Fig. 5).

## Discussion

Integrating in silico predictions with in vivo studies, our work indicates that *C. elegans* DRH-1/RLR contains signaling-competent tandem CARDs that can activate a transcriptional immune response. In the absence of a pathogen trigger, we demonstrated that intestine-specific overexpression of DRH-1(2CARD) promotes IPR activation, partially dependent on the transcription factor *zip-1* (Figs. 1 and 2). IPR activation by DRH-1(2CARD) conferred increased resistance to viral infection and increased survival following heat shock (Fig. 3), which are both phenotypes that have been previously linked to IPR activation (9). Prior to this study, the tissue requirement for DRH-1 in the context of Orsay virus infection remained unclear, although recent findings support a role for DRH-1 acting in the intestine to combat age-related pathology (44). Our findings here with both tissue-specific expression (Figs. 1 and 3 and *SI Appendix*, Fig. S3) as well as tissue-specific knock-down (Fig. 4) support a model in which DRH-1 functions in the intestine to induce immune gene activation upon viral infection. The intestine-specific requirement for DRH-1 is consistent with the observation that Orsay virus exhibits tropism for intestinal tissue (45). Subcellular analysis of virus-infected intestinal cells reveals the presence of cytoplasmic DRH-1 puncta that colocalize with dsRNA (Fig. 5). Furthermore, domain analysis suggests that the helicase and C-terminal domain mediate DRH-1 puncta formation (*SI Appendix*, Fig. S7). Overall, our findings suggest that DRH-1 signals through an N-terminal 2CARD in intestinal cells to induce the IPR during viral infection.

CARD-containing proteins have long been known to be important in mammalian immunity for activating the IFN-I response (e.g., CARDs in RLRs) (16), as well as for activating inflammasome formation and cell death (e.g., CARDs in NLRs) (46), and more recently for coordinating defense in nonmammalian systems. As described in a recent preprint, CARD-like domains were found to mediate cell death during phage infection as part of an immune defense system in the bacteria *Lysobacter enzymogenes* (47). Results from that study suggest that CARDs may constitute evolutionarily conserved immune signaling modules that likely originated from bacteria and are retained in mammals. In *C. elegans*, structural and functional analyses have described a role for CARDs in facilitating interactions between cell death components CED-3(CEll Death abnormality)/caspase and CED-4/Apaf-1 to regulate apoptosis (48). Although apoptosis does not seem to have a prominent role in *C. elegans* immunity, CED-3/caspase and CED-4/Apaf-1 have been implicated in the restriction of viral replication in a vaccinia virus infection model (49). In the context of RLRs in *C. elegans*, crystal structures of DRH-3 have recently revealed 2CARDs at the N terminus (27). While DRH-3 is a component of the antiviral RNAi pathway, the signaling role of DRH-3(2CARD) remains to be determined. Notably, previous domain analysis of DRH-1 demonstrated that the NTD (2CARD) was required for reducing levels of viral RNA (12). In the same study, however, the authors found that expression of DRH-1 NTD was not sufficient to reduce viral RNA levels, which may be due to differing assays and strains used in that study compared to ours. Further investigation into the role of CARDs in *C. elegans* immunity may reveal a better understanding of mechanisms that govern antiviral signaling, as well as the formation of signaling complexes, in an invertebrate host.

Formation of classical RLR signaling complexes requires binding of RNA ligands, such as dsRNA and 5′ triphosphorylated RNA, to initiate protein oligomerization via CARD-CARD interactions (16). Signaling-competent oligomers then interact with CARDs found in MAVS, a mitochondrially localized signaling factor, to initiate a downstream signaling cascade (50). Although *C. elegans* lacks a known MAVS homolog, our subcellular localization analyses here indicate that DRH-1 forms puncta upon viral infection (Fig. 5), which may reflect protein oligomerization at a signaling hub, similar to what is observed in mammals. Notably, however, recent findings suggest that RLR signaling occurs prior to oligomerization (51). In addition, the observation that DRH-1 colocalizes with dsRNA antibodies inside virus-infected cells supports a model in which DRH-1 binds viral replication products or modified host RNAs generated during infection. While recent in vitro work has revealed that DRH-1 can bind blunt-end dsRNA, the native ligand for DRH-1 remains unknown (17). Furthermore, whether the dsRNA observed in our study originates from the host or the virus remains to be determined.

Canonical RLR signaling in mammals involves MAVS filament formation, as described above, followed by activation of the TANK-binding kinase 1 (TBK-1) kinase, then phosphorylation and nuclear localization of the IRF3 transcription factor, which induces IFN-I gene expression and a systemic innate immune response (16). *C. elegans* lacks known homologs of TBK-1, IRF3 as well as MAVS, so its downstream signaling mechanisms are unknown (9). ZIP-1 is the only signaling factor identified so far that activates transcription downstream of DRH-1. ZIP-1 belongs to a branch of the bZIP transcription factor family that expanded in *C. elegans* (52) and does not have an obvious mammalian ortholog (23). In this study, we found that ZIP-1 was partially required for DRH-1(2CARD) signaling, but it did not localize to the nucleus, in contrast to previously described nuclear translocation of ZIP-1 seen upon viral infection or other IPR triggers. In the endogenously tagged GFP strain used for our study, it might be that visible nuclear localization of ZIP-1 may require other triggers, or a trigger stronger than DRH-1(2CARD). Nonetheless, the partial requirement for ZIP-1 is consistent with prior studies (23), including those demonstrating that the IPR can be a systemic immune response in *C. elegans* (32), similar to the antiviral IFN-I response in mammals.

A feature of systemic immune responses includes cell nonautonomous signaling across different tissues. In our study, we investigate the effects of intestine-specific DRH-1(2CARD) overexpression on pathogen resistance. Our findings that DRH-1(2CARD) overexpression confers resistance against virus but not *N. parisii* is somewhat surprising given the upregulation of IPR genes in DRH-1(2CARD) animals. One potential explanation for this difference is that resistance against *N. parisii* may require IPR signaling from other tissues. For example, epidermal-to-intestinal signaling has been previously shown to play a salient role in IPR-mediated immune defense against *N. parisii* (32). Alternatively, it is possible that, in addition to the tissue-specificity of IPR gene expression, resistance to *N. parisii* also depends on nontranscriptional responses or on the transcriptional induction of other infection-related genes. Future work can explore the transcriptional and functional outcomes of DRH-1(2CARD) overexpression in other tissues, including the effects on resistance to *N. parisii* infection.

In recent years, the study of immunity in diverse hosts has revealed remarkable evolutionary conservation in immune signaling pathways from eukaryotes to bacteria (1). Our findings expand upon prior work, which identified a role for DRH-1 in activating a transcriptional immune response (20), to uncover insights into shared characteristics of RLR-mediated signaling between *C. elegans* and mammals. A hallmark of antiviral immunity in mammals is the induction of systemic immunity triggered by secreted IFN-I ligands signaling through IFN receptors, which are ostensibly absent in *C. elegans*, although other ligand–receptor systems likely mediate systemic immunity as part of the IPR (32). Our work, however, supports the notion that RLR activation of the IFN-I response in mammals, and the IPR in *C. elegans*, resulted from divergent evolution of an ancient immune pathway for sensing cytosolic nucleic acid (9). Proteins involved in coevolutionary host/pathogen battles commonly undergo amino acid sequence diversification (53). Therefore, the absence of sequence-based homologs does not necessarily indicate the absence of conservation. For example, the *Vibrio cholera* cGAS homolog (*dncV*) and human cGAS share high structural and functional similarity despite only sharing 10% primary sequence identity (54). Future efforts aimed at identifying signaling proteins downstream of DRH-1 in the *C. elegans* RLR pathway, such as determining whether there is a MAVS-like protein or a distinct downstream signal, may identify novel antiviral signaling factors involved in innate immune defense.

## Materials and Methods

The predicted protein structure of DRH-1 was obtained from the AlphaFold Protein Structure Database (http://alphafold.ebi.ac.uk/) (24, 25). *C. elegans* strains (*SI Appendix*, Table S1) were maintained on Nematode Growth Media agar plates containing streptomycin-resistant *Escherichia coli* OP50-1. All constructs and primers used in this study are listed in *SI Appendix*, Tables S2 and S3, respectively. Quantification of *pals-5::*GFP and *F26F2.1p::*GFP reporter fluorescence was performed in Fiji or analyzed on a Copas Biosort (Union Biometrica). All subcellular imaging was performed using a Zeiss LSM700 confocal microscope with Zen 2010 software. All infection assays were performed on developmentally synchronized animals. Orsay virus infections were performed using virus from the same batch of virus filtrate, which was prepared as previously described (28). For microsporidia infections, *N. parisii* spores were prepared as previously described (55). Tissue-specific RNAi was performed via the feeding method. The anti-dsRNA antibody clone rJ2 (Sigma-Aldrich) was used for dsRNA localization studies. All statistical analyses were performed in R. A detailed description of all methods used in this study can be found in *SI Appendix*.

## Supplementary Material

Appendix 01 (PDF)

Dataset S01 (XLSX)

Dataset S02 (XLSX)

## Data Availability

All study data are included in the article and/or supporting information.
