# Supplementary material for: Caenorhabditis elegans RIG-I-like receptor DRH-1 signals via CARDs to activate antiviral immunity in intestinal cells
Source: Proc Natl Acad Sci U S A. 2024 Jul 9;121(29):e2402126121. doi: 10.1073/pnas.2402126121 (PMC11260149; doi:10.1073/pnas.2402126121)
Supplement: Supplementary file 1 — Appendix 01 (PDF) [file pnas.2402126121.sapp.pdf]

PNAS

Supporting Information for
C. elegans RIG-I-like receptor DRH-1 signals via CARDs to activate anti-viral immunity in intestinal cells

Lakshmi E. Batachari, Alyssa Y. Dai, Emily R. Troemel

Emily R. Troemel
Email: etroemel@ucsd.edu

This PDF file includes:

Supporting text
Figures S1 to S7
Tables S1 to S3
SI References

Other supporting materials for this manuscript include the following:

Datasets S1 to S2

Supporting Information Text

Extended Materials and Methods

In silico protein structure analyses

The predicted protein structure of DRH-1 was obtained from the AlphaFold Protein Structure Database (http://alphafold.ebi.ac.uk/) (1, 2). RIG-I CARD domains (PDB ID: 4p4h) were superimposed onto the predicted structure of DRH-1 in PyMOL. PyMOL was also used to isolate the putative DRH-1 NTD (first 276 amino acids), which was queried against known structures in the PDB database using Foldseek (http://search.foldseek.com/search) (3) and Dali (http://ekhidna2.biocenter.helsinki.fi/dali/) (4, 5) protein structure comparison platforms.

C. elegans Maintenance

C. elegans strains were maintained on Nematode Growth Media (NGM) agar plates containing streptomycin-resistant *Escherichia coli* OP50-1, unless otherwise specified. S1 Table lists all strains used in this study.

Molecular cloning and transgenic *C. elegans* strains

Unless indicated otherwise, all constructs are expressed in an RNAi-deficient *rde-1* mutant background to reduce transgene silencing. For cloning *drh-1(2CARD)::mScarlet,* a cDNA sequence encoding the first 276 amino acids of *drh-1*, in addition to a C-terminal sequence-optimized mScarlet tag (also referred to as wrmScarlet) (6), was synthesized as a HiFi gBlock (Integrated DNA Technologies). The gBlock also included 50 base pair (bp) 5' and 3' homology to intestine-specific promoter *vha-6p* and *unc-54* 3'utr, respectively. Primers LEB001 and LEB002 were used to amplify off of template plasmid pET636 to generate a vector backbone containing *vha-6p,* an *unc-54* 3'utr, a 3xFLAG tag, and an ampicillin resistance cassette. The *drh-1(2CARD)::mScarlet* sequence was inserted into the vector backbone by Gibson assembly to generate the final construct pET770 [*vha-6p::drh-1(2CARD)::mScarlet::3xFLAG::unc-54 3'utr*]. Gibson assembly was performed using the NEBuilder HiFi DNA Assembly kit (New England Biolabs). 100 ng/µl of pET770 was co-injected along with a body wall muscle co-injection marker (17 ng/µl) into *jyIs8; rde-1* animals to create extrachromosomal array transgenic line *jyEx302* (ERT1076). Because DRH-1(2CARD)::mScarlet fluorescence was visible, additional transgenic lines were created without the use of a co-injection marker. In particular, extrachromosomal array lines *jyEx304* (ERT1207) and *jyEx305* (ERT1182) weLre generated by injecting 100 ng/µl of pET770 without a co-injection marker.

Figure 1C and 1D demonstrate that DRH-1(2CARD)::mScarlet transgenic lines *jyEx302*, *jyEx304* and *jyEx305* all induce the pals-5p::GFP reporter. However, specific lines were used for specific subsequent experiments for the following reasons. First, developmental analysis of *pals-5p::*GFP

expression (Fig. 1E) was performed with transgenic line *jyEx302*, as this was the first line DRH-1(2CARD) line established. Next *jyEx305* was chosen for qRT-PCR (Fig. 1F), because it has the highest rate of transmission of the extrachromosomal array, increasing the percentage of transgenic animals in a large mixed population used to obtain enough RNA for analysis. This strain also has the lowest expression of mScarlet among all three transgenic lines, and for this reason we chose *jyEx305* for Fig. 2 and S2, to minimize red fluorescence bleed-through into the green channel, in order to better image *pals-5p::*GFP and *ZIP-1::*GFP. All three lines increased resistance to viral infection (Fig. 3A, B), but only transgenic line *jyEx304* was used for subsequent *N. parisii* infection and thermotolerance assays, as this line showed significantly decreased viral load, but also exhibited less developmental delay compared to *jyEx305*. Localization of DRH-1(2CARD) was assessed in transgenic line *jyEx304* (Fig S4A, B) as this line showed significantly decreased viral load (Fig. 3B) while still providing a sufficient number of infected transgenic animals for analysis.

To create the empty vector control, the *drh-1(2CARD)* sequence was deleted from pET770 using the Q5 Site-Directed Mutagenesis Kit (New England Biolabs). Primers LEB003 and LEB004 were used to amplify around the *drh-1(2CARD)* sequence in pET770. The linearized fragment was subsequently recircularized and transformed into chemically-comptent NEB DH5α cells (New England Biolabs) to generate the resulting plasmid pET786 [*vha-6p::mScarlet::unc-54 3'utr*]. 10 ng/µl of pET786 was injected into *jyIs8; rde-1* animals. Of note, the negative control construct was initially injected at 100 ng/µl, which is the injection concentration used for pET770 (see above). However, injections at this concentration resulted in extremely high levels of mScarlet expression, in which red fluorescence levels greatly exceeded that of DRH-1(2CARD)::mScarlet lines. Additionally, bleed-through of red fluorescence into the green channel used for imaging was observed in these animals, impairing assessment of *pals-5p::GFP* expression. Therefore, a decreased injection concentration of 10 ng/µl, was necessary to achieve protein expression levels comparable to that of DRH-1(2CARD)::mScarlet, as well as mitigate any off-target effects due to extremely high concentrations of mScarlet. Of note, the mScarlet expression in the negative control line was still higher than in the DRH-1(2CARD) lines. Filler DNA (pBluescript) was added to the injection mix to reach a final DNA concentration of 100 ng/µl.

For intestinal expression of full-length *drh-1,* an intron-containing sequence for *drh-1* was amplified from plasmid Tian233 using primers LEB005 and LEB006, which contain a 40 bp 5' to *vha-6p* and a 36 bp 3' homology to *mScarlet*. A vector backbone was generated by linearization of pET770, and PCR products were assembled using the NEBuilder HiFi DNA Assembly kit (New England Biolabs) to generate the final construct pET788. 100 ng/µl of pET788 was injected into *jyIs8; rde-1* animals.

For intestinal expression of the *drh-1* helicase and CTD, the region encoding *drh-1(2CARD)* was deleted from pET788 (full-length *drh-1* overexpression construct) using the Q5 Site-Directed Mutagenesis Kit (New England Biolabs). Primers LEB007 and LEB008 were used to amplify around the *drh-1(2CARD)* sequence in pET788. The linearized fragment containing the *drh-1(HC)* region was subsequently recircularized and transformed into chemically-comptent NEB DH5α cells (New England Biolabs) to generate the resulting plasmid pET794 [*vha-6p::drh-1(HC)::mScarlet::unc-54 3'utr*]. 100 ng/µl of pET794 was injected into *jyIs8; rde-1* animals.

All plasmid sequences were validated by whole-plasmid sequencing. Constructs used in this study can be found in S2 Table. Primer sequences are listed in S3 Table.

Synchronization of *C. elegans*

To synchronize development, gravid adult animals were washed off plates with M9 media and transferred into a 15 ml conical tube. Worms were pelleted at 3,000 rpm for 30 sec and resuspended in 3 ml of M9 and 1 ml of bleaching solution (500 µl of 5.65–6% sodium hypochlorite solution and 500 µl of 2 M NaOH). After most adults had dissolved, released embryos were washed five times with 15ml of M9 and resuspended in a final volume of 3 ml of M9. Embryos were incubated at 20 ºC with continual rotation for 20-24 h to hatch L1s.

***pals-5p::*GFP and *F26F2.1::*GFP fluorescence measurements**

DRH-1(2CARD) strains were synchronized by bleaching and grown at 20 ºC for 24 h (L2/L3 larval stage), 44 h (L4 larval stage), or 68 h (adults). Worms were washed off plates, resuspended in M9, and anesthetized with 10 µM levamisole prior to imaging in a 96-well plate format on a ImageXpress Nano plate reader using the 4x objective (Molecular Devices, LLC, San Jose, CA). Background-corrected mean fluorescence intensity was quantified in Fiji by tracing the worm area and calculating the mean fluorescence intensity (MFI). To correct for background fluorescence, MFI of the well was subtracted from the MFI of each worm. For tissue-specific RNAi experiments, GFP fluorescence was measured using a COPAS Biosort instrument (Union Biometrica).

RNA isolation and qRT-PCR

For DRH-1(2CARD) lines in Fig. 1F, 2C, S2C, and S4C, 40 adults were transferred to 10-cm NGM plates due to smaller brood sizes. For non-transgenic controls, 20 adults were transferred to 10-cm NGM plates. As mentioned above, DRH-1(2CARD) lines were generated in a RNAi-defective *rde-1* mutant background to enhance transgene expression. Thus, non-transgenic control strains are either *rde-1* mutants with (Fig. S1B) or without (Fig. 1F) the *pals-5p::GFP* reporter. Two replicates (two plates) were set up per strain and worms were incubated for 96 h at 20 ºC to produce progeny. RNA was isolated from these progeny (thousands of animals) using TRI Reagent and 1-bromo-3-chloropropane (BCP) (Molecular Research Center) followed by isopropanol and ethanol

washes. Pure RNA was resuspended in nuclease free water. Following cDNA synthesis using the iScript synthesis kit (Bio-Rad), qPCR was performed with iQ SYBR Green Supermix (Bio-Rad) on a CFX Connect Real-Time PCR Detection System (Bio-Rad). In Fig. S4B, RNA was isolated 24 h after infecting synchronized *rde-1* L4s with either Orsay virus or *N. parisii* at 25ºC. For measurements of *drh-1* transcript levels in Fig. S5, synchronized *jyIs8* L1s were plated on control or *drh-1* RNAi plates and grown at 20ºC for 44 h following the protocol detailed below. L4 animals were exposed to virus for 24 h prior to RNA isolation and cDNA synthesis following the steps above.

For all qPCR analyses, relative gene expression ratios were determined by the Pfaffl method (7). Primer efficiencies were calculated by generating standard curves. Gene expression values were normalized to the expression of housekeeping gene *snb-1*. qRT-PCR primer sequences are listed in S3 Table.

Bortezomib treatment

Proteasome inhibition by bortezomib (Selleck Chemicals) was performed as previously described (8). Synchronized L1 animals were plated on 6-cm NGM plates containing OP50-1 and grown for 44 h at 20 ºC. Bortezomib in DMSO was top-plated to reach a final concentration in the agar of 20 µM. DMSO was used as a vehicle control. Plates were then incubated for 4 h at 20 ºC prior to imaging. To assess ZIP-1::GFP localization, animals were anesthetized with 100 µM levamisole and imaged on a Zeiss LSM700 confocal microscope with Zen 2010 software.

Orsay virus infections

All infections were performed using virus from the same batch of virus filtrate, which was prepared as previously described (9). Animals were infected with virus filtrate at the L1 or L4 stage. A minimum of two replicates (two plates) were set up per genotype per experiment. At least three independent experiments were performed per timepoint. For L1 infections, developmentally synchronized animals were exposed to a mixture of OP50-1 bacteria, M9, and virus filtrate for 18 h at 20 ºC (Fig 3A, B). For L4 infections, synchronized animals were exposed to a mixture of OP50-1 bacteria, M9, and virus filtrate for 24 h at 20 ºC (Fig 4, 5). For infection rate measurements and localization analyses, animals were washed in M9 and fixed in 4% paraformaldehyde for 15 min. Following fixation, worms were incubated at 47 ºC overnight with fluorescein (FAM)- or Quasar 670-conjugated FISH probes that target Orsay virus RNA1 and RNA2 (Biosearch Technologies). Infection rate was assessed using a Zeiss AxioImager M1 compound microscope. For each experimental replicate, a minimum of 100 animals per genotype were scored for the presence of FISH fluorescent signal. For subcellular localization analyses, animals were imaged using a Zeiss LSM700 confocal microscope with Zen 2010 software. For tissue-specific knockdown experiments, animals were anesthetized with 10 µM levamisole prior to analysis using a COPAS Biosort

instrument (Union Biometrica) to measure fluorescence. For qRT-PCR analysis, see RNA isolation and qRT-PCR section.

Microsporidia infections

N. parisii spores were prepared as previously described (10). A mixed population of DRH-1(2CARD) animals and non-transgenic siblings was synchronized at the L1 stage and plated on 6-cm NGM plates along with a mixture of OP50-1, M9, and one million spores for 30 h at 25ºC. A minimum of four replicates (four plates) were set up per treatment. Three independent experiments were performed. To assess the presence of *N. parisii* meronts, animals were washed in M9 and fixed in 4% paraformaldehyde for 15 min prior to an overnight incubation at 47 ºC with a FAM-conjugated FISH probe that hybridizes to *N. parisii* ribosomal RNA (Biosearch Technologies). Samples were sorted based on red fluorescence using the COPAS Biosort instrument (Union Biometrica) to obtain homogenous populations of either DRH-1(2CARD) animals or non-transgenic siblings. Green fluorescence was measured for each population using the COPAS Biosort instrument (Union Biometrica). For each genotype, the median green fluorescence of the infected population was normalized to the median fluorescence of the uninfected population. For qRT-PCR analysis, see RNA isolation and qRT-PCR section.

Development rate measurements

60 gravid adults were transferred onto a 10-cm NGM plate containing OP50-1 and incubated at 20 ºC for 2 h. Adults were gently washed off of plates with M9 such that only eggs remained on the plate, and eggs were incubated at 20 ºC for 48 h and 64 h. For each timepoint, development rate was determined by scoring the percentage of L4 stage or older animals. 100 animals were scored per genotype across three independent experimental replicates.

Thermotolerance assays

Thermotolerance phenotypes were assayed as previously described (11). For each genotype, L4 animals (three plates; 30 L4s per plate) were transferred to NGM plates containing OP50-1. Animals were exposed to heat-shocked for 2 h at 37.5 ºC in a dry incubator. Immediately following heat shock, plates were transferred to room temperature for 30 min. followed by incubation at 20 ºC for 24 h. Survival was scored by assessing mobility, wherein dead worms were identified by failure to respond to a single touch using a worm pick. Three independent experimental replicates were performed on different days.

Tissue-specific RNA interference

Systemic, intestine-specific, and epidermis-specific RNAi was performed via the feeding method. RNAi bacterial clones (*drh-1* and L4440 empty vector control) were inoculated in 5 ml LB containing

50 µg/ml carbenicillin and incubated in a 37 ºC shaking incubator (250 rpm) for 16 h. Overnight cultures were seeded onto NGM plates supplemented with 5 mM IPTG and 1 mM carbenicillin. Seeded RNAi plates were incubated at room temperature for 4 days. Synchronized L1 animals were transferred to RNAi plates and grown at 20 ºC for 44 h. Animals were then exposed to virus for 24 h and then analyzed for *pals-5p::*GFP expression on a COPAS Biosort instrument (Union Biometrica), as described above.

Quantification of puncta

QuPath image analysis software (12) was used to quantify subcellular localization patterns of DRH-1(2CARD) and DRH-1(HC) during viral infection. The polygon tool was used to annotate an intestinal region encompassing two nuclei within each animal. The number of puncta within the annotated region was determined using automated cell detection with the following parameters; "requestedPixelSizeMicrons": 0.2, "backgroundRadiusMicrons: 10.0, "backgroundByReconstruction": true, "medianRadiusMicrons": 1.0, "sigmaMicrons": 0.4, "minAreaMicrons": 1.0, "maxAreaMicrons": 40.0, "threshold": 9.0, "watershedPostProcess": true, "cellExpansionMicrons": 0.0, "includeNuclei": true, "smoothBoundaries": true, "makeMeasurements": true. For detection of aggregates, the cutoffs for minimum area ("minAreaMicrons") and maximum area ("maxAreaMicrons") were set to 40 and 200, respectively. Puncta/aggregate density measurements were calculated by dividing the number of puncta/aggregates by the total area of the annotated region for each animal.

Immunohistochemistry

For dsRNA localization analyses, synchronized L1s were plated onto 10-cm NGM plates and grown for 44 h at 20 ºC to reach the L4 stage. Animals were then exposed to virus filtrate for 24 h at 20 ºC, as described above. After 24 h, adults were washed off of plates with M9 and anesthetized in 100 µM levamisole. Intestines were dissected out of 100-200 adults and fixed in 4% paraformaldehyde for 15 min. To visualize virus-infected cells, samples were incubated with Quasar 670-conjugated FISH probes that hybridize to Orsay virus RNA1 and RNA2 (Biosearch Technologies). Following a 6-8 h incubation at 47 ºC, FISH probes were washed off and dissected intestines were incubated in block buffer (PBS, 0.5% Tween-20, 1 mM EDTA, 5% BSA, 0.05% NaN_3) overnight at 4 ºC. Dissected intestines were then stained with a 1:200 dilution of the anti-dsRNA antibody clone rJ2 (Sigma-Aldrich) for 2-4 h, followed by 10 µg/ml of goat anti-mouse IgG(H+L) cross-adsorbed secondary antibody conjugated to Alexa Fluor 488 (Invitrogen) for 1-2 h. Staining was performed in block buffer at room temperature.

Statistics

All statistical analyses were performed in R. Q-Q plots were used to assess normality of the data, and parametric tests were used when appropriate. A nonparametric test was applied to data that did not meet the assumptions for a parametric test.

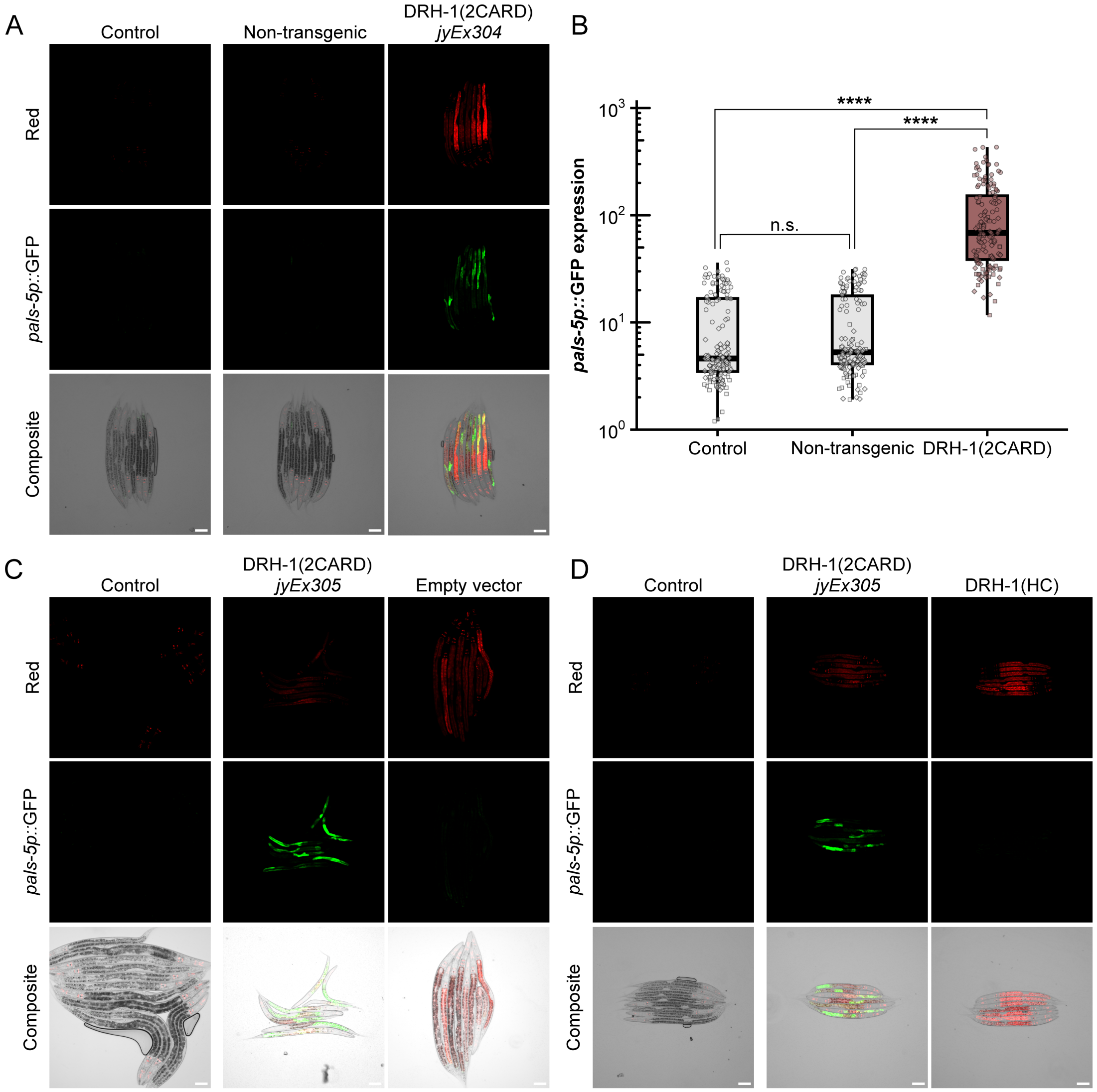

Fig. S1. DRH-1(2CARD) specifically induces *pals-5p::*GFP*.* (A) Representative images of DRH-1(2CARD) animals from line *jyEx304,* non-transgenic siblings, and control animals originally used to generate the transgenic line. Scale bar = 50 µm. (B) Quantification of *pals-5p*::GFP in (A)*.* Quantification of non-transgenic siblings was performed using the same images that were used for analysis in Fig. 1D. Data for the control and DRH-1(2CARD) animals are the same as that shown in Fig. 1D. Horizontal lines in box-and-whisker plots represent median values, and the box reflects the 25th to 75th percentiles. A Kruskal-Wallis test followed by Dunn's post-hoc was used to determine p-values; ****$p < 0.0001$. Each dot represents an individual animal; 150 animals were analyzed across three independent experiments for genotype; ****$p < 0.0001$. (C) Representative images showing *pals-5p::*GFP induction in DRH-1(2CARD) transgenic animals (line *jyEx305*) and absence of *pals-5p*::GFP induction in empty vector control (line *jyEx336*). Scale bar = 50 µm. (D) Representative images showing the absence of *pals-5p*::GFP induction in DRH-1(HC) transgenic animals. Scale bar = 50 µm. All animals shown in (A), (C), and (D) constitutively express *myo-2p::*mCherry in the pharynx as part of the *jyIs8[pals-5p::gfp]* transgene.

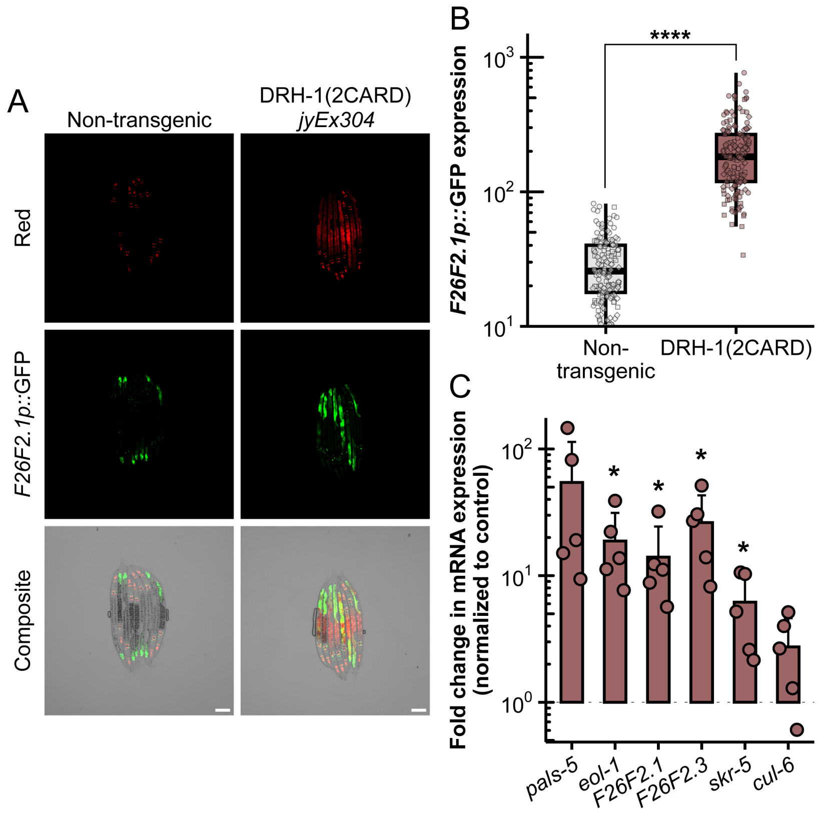

Fig. S2. DRH-1(2CARD) induces *F26F2.1p*::GFP and endogenous IPR gene expression. (A) Representative images showing increased *F26F2.1p*::GFP expression in DRH-1(2CARD) transgenic animals relative to their non-transgenic siblings. Scale bar = 50 µm. (B) Quantification of *F26F2.1p*::GFP in DRH-1(2CARD) animals and non-transgenic siblings shown in (A). Horizontal lines in box-and-whisker plots represent median values, and the box reflects the 25th to 75th percentiles. Each dot represents an individual animal; 150 animals were analyzed across three independent experiments for each strain. A Mann-Whitney *U* test was used to determine significance; ****p < 0.0001. (C) qRT-PCR analysis of DRH-1(2CARD) line *jyEx305* (expresses *pals-5p::*GFP reporter) shown in. RNA was extracted from a mixed-stage population containing both DRH-1(2CARD) transgenic animals and their non-transgenic siblings. Fold change in gene expression was determined relative to a non-transgenic control strain (*rde-1* mutant in a *pals-5p::*GFP background). Bars represent the mean across experimental replicates; error bars represent the standard deviation. Each dot represents a biological replicate (a plate with a minimum of 2000 animals); four independent experimental replicates were performed. A one-tailed *t*-test was used to calculate p-values; *p < 0.05.

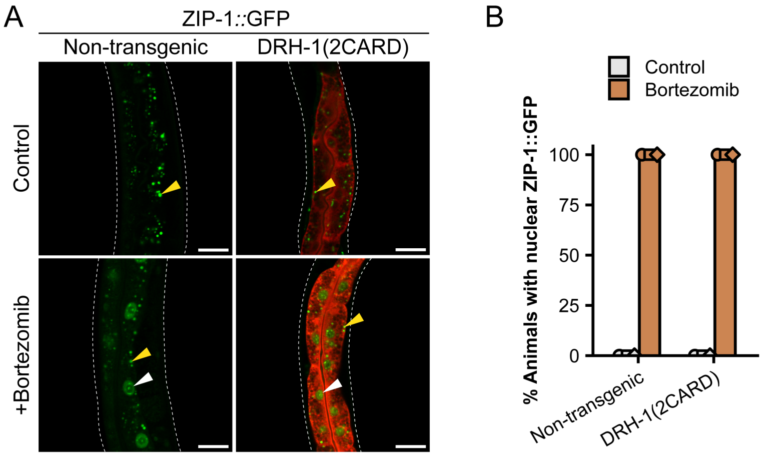

Fig. S3. DRH-1(2CARD) expression does not promote nuclear localization of ZIP-1::GFP. (A) Representative images showing ZIP-1::GFP expression in DRH-1(2CARD) transgenic animals and non-transgenic siblings. ZIP-1::GFP is not visible in the nuclei of DRH-1(2CARD) animals. Bortezomib treatment was used as a positive control for nuclear localization of ZIP-1::GFP. White arrowheads indicate ZIP-1::GFP expression in the nucleus. Yellow arrowheads indicate autofluorescence from intestinal gut granules. Scale bar = 25 μm. (B) ZIP-1::GFP is not present in intestinal nuclei of untreated animals, but is expressed in 100% of animals treated with bortezomib. Localization pattern of ZIP-1::GFP is the same for both DRH-1(2CARD) animals and non-transgenic siblings. For each genotype and treatment, 45 total animals were scored. Bars represent the mean across biological replicates; error bars represent the standard deviation. Each dot represents a biological replicate (a plate with 15 animals).

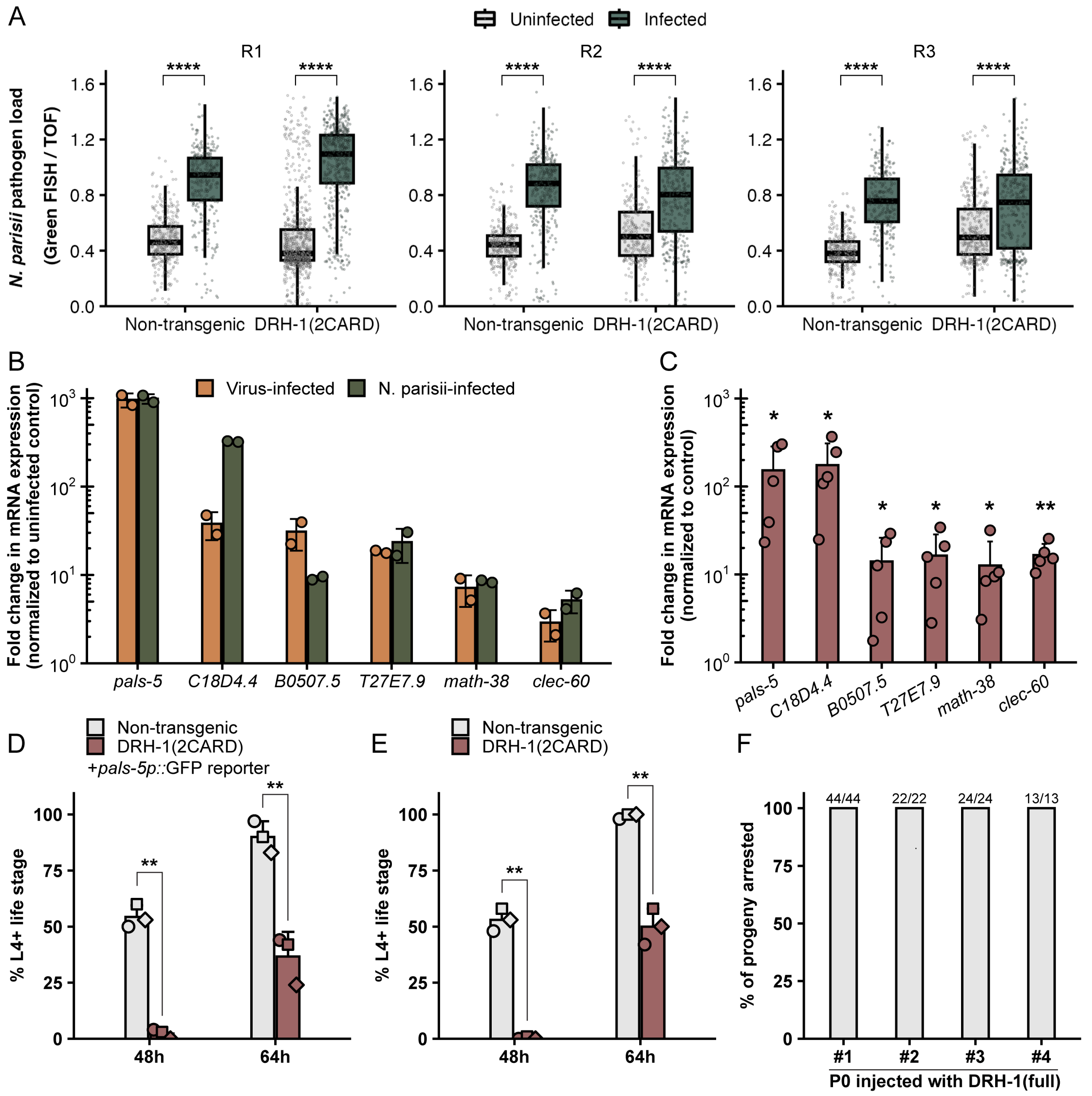

Fig. S4. DRH-1(2CARD) expression does not reduce *N. parisii* pathogen load and delays development. (A) Quantification of *N. parisii* pathogen load in individual experiments (combined results shown in Fig. 3D) by FISH using fluorescein-conjugated (green) probes that target *N. parisii* ribosomal RNA. A COPAS Biosort instrument was used to measure green fluorescence normalized to time-of-flight (a measure of worm size). Dots represent individual animals. Left (R1): n = 353 (non-transgenic; uninfected), 317 (non-transgenic; infected), 791 (DRH-1(2CARD); uninfected), or 535 (DRH-1(2CARD); infected). Center (R2): n = 336 (non-transgenic; uninfected), 337 (non-transgenic; infected), 260 (DRH-1(2CARD); uninfected), or 385 (DRH-1(2CARD); infected). Right (R3): n = 322 (non-transgenic; uninfected), 271 (non-transgenic; infected), 384 (DRH-1(2CARD); uninfected), or 372 (DRH-1(2CARD); infected). Horizontal lines in box-and-whisker plots represent median values, and the box reflects the 25th to 75th percentiles. Each panel displays data from an independent experimental replicate. A Mann-Whitney *U* test was used to calculate p-values; ****p < 0.0001. (B) qRT-PCR analysis of genes, identified in a published RNAseq dataset (13), that are likely to be induced during *N. parisii* but not Orsay virus infection. Animals were infected with either Orsay virus or *N. parisii* at 25ºC for 24 h. Bars represent the mean across biological replicates; error bars represent standard deviation. Each dot represents a biological replicate (a plate with 2000 animals). (C) Expression of genes in (B) upon overexpression of DRH-1(2CARD). qRT-PCR

analysis was performed on a mixed population of DRH-1(2CARD) animals from line *jyEx305.* Bars represent the mean across experimental replicates; error bars represent standard deviation. Each dot represents a biological replicate (a plate with a minimum of 2000 animals); four independent experimental replicates were performed. A one-tailed *t*-test was used to calculate p-values; *$p < 0.05$, **$p < 0.01$. (D) DRH-1(2CARD) animals exhibit delayed development relative to non-transgenic siblings in a strain background with (D) or without (E) the *pals-5p::*GFP reporter. Bars represent the mean across experimental replicates; error bars represent the standard deviation. Each dot represents an experimental replicate. A two-tailed *t*-test was used to calculate p-values; **$p < 0.01$. (F) Intestinal overexpression of full-length DRH-1 leads to developmental arrest at L1/L2 larval stages in 100% of transgenic progeny across four injections. The fraction of transgenic progeny exhibiting larval arrest is displayed above each bar.

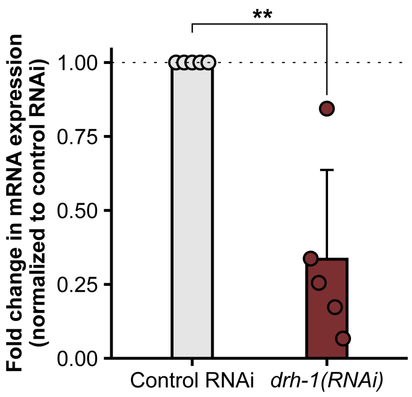

Fig. S5. RNAi against *drh-1* results in decreased *drh-1* mRNA transcript levels. qRT-PCR analysis of *drh-1* confirms significantly decreased mRNA expression following RNAi against *drh-1*. Bars represent the mean across experimental replicates; error bars represent standard deviation. Each dot represents an experimental replicate; five independent experimental replicates were performed. A one-tailed *t*-test was used to determine significance; $^{**}p < 0.01$.

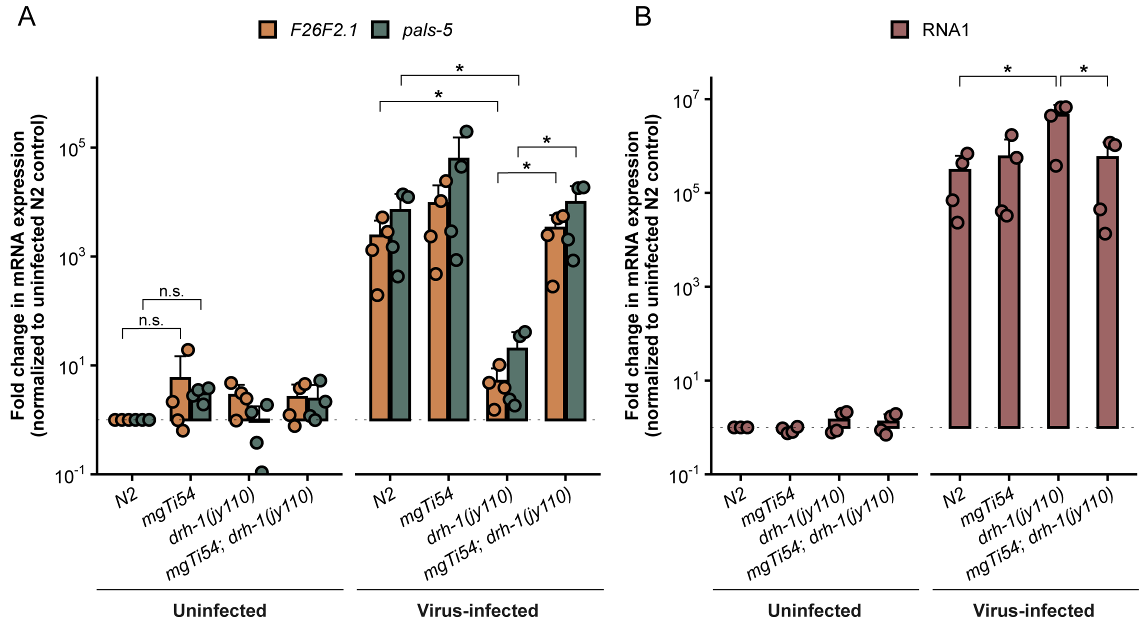

Fig. S6. Expression of the *rpl-28p::mScarlet::drh-1* transgene rescues *pals-5* expression and Orsay RNA1 levels upon viral infection. (A) During viral infection, expression of the mScarlet::DRH-1 transgene (*mgTi54)* in a *drh-1(jy110)* deletion mutant is sufficient to rescue mRNA levels of (A) IPR genes *F26F2.1* and *pals*-5, as well as (B) Orsay RNA1 to WT levels. qRT-PCR analysis of *F26F2.1*, *pals-5*, and Orsay RNA1 in WT animals (N2), a *drh-1(jy110)* deletion mutant, and animals that express the *mgTi54* transgene in a *drh-1(jy110)* or WT background. Fold change in gene expression was determined relative to the uninfected WT (N2) control. Bars represent the mean; error bars represent the standard deviation. Each dot represents a biological replicate (a plate with 2000 animals); three independent experimental replicates were performed. A one-sample Wilcoxon signed rank test was used to compare the distribution of values against a hypothetical value of 1 in the uninfected group. A Mann-Whitney *U* test was used to calculate p-values for comparisons between samples in the infected group; *$p < 0.05$.

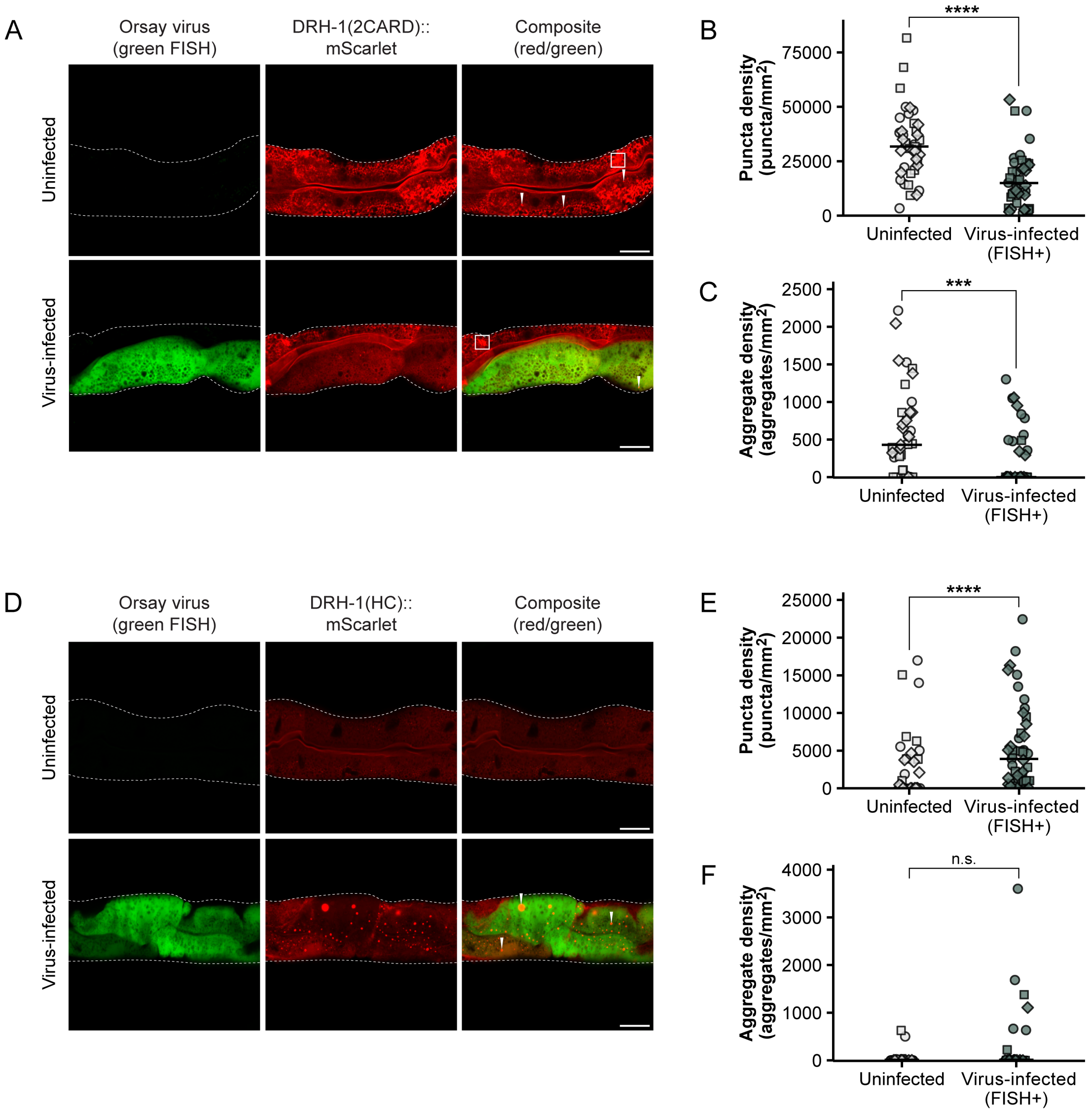

Fig. S7. DRH-1 helicase and C-terminal domain, DRH-1(HC), mediates puncta formation during viral infection. (A) Representative images showing DRH-1(2CARD)::mScarlet localization in uninfected and virus-infected intestinal cells. Intestine is outlined by white dashed line. In the absence of infection, animals exhibit DRH-1(2CARD) aggregates in the intestinal cytoplasm (white box), with some animals exhibiting both DRH-1(2CARD) aggregates and puncta (white arrowhead). FISH-positive cells in infected animals (n = 43) show decreased DRH-1(2CARD) puncta density (B) and aggregate density (C) compared to cells in uninfected animals (n = 41). Density measurements are determined relative to the area of the intestinal region analyzed. (D) Representative images showing DRH-1(HC)::mScarlet localization in uninfected and virus-infected intestinal cells. Intestine is outlined by white dashed line. Intestinal cells within uninfected animals (n = 45) exhibit minimal DRH-1(HC) puncta and aggregate formation. In virus-infected animals (n = 45), FISH-positive cells exhibit significantly increased DRH-1(HC) puncta density (B), but aggregate density is not significantly different from the control. For (B), (C), (E), and (F), horizontal bars represent the median across three experimental replicates; dots represent individual animals.

Table S1. List of strains used in this study.

Strain name	Genotype (transgene or mutant alelle details)	Figures	Source	Notes
ERT054	*jyIs8[pals-5p::gfp, myo-2p::mCherry] X*	4, S5	(9)	
ERT1053	*mgTi54[rpl-28p::mScarlet::drh-1]; drh-1(jy110) IV*	5A/B, S6	(14)	GR3325 from Mao et al., 2020 was crossed with *drh-1(jy110)* in this study
ERT1076	*jyEx302[vha-6p::drh-1-NTD::wrmScarlet::3xFLAG::unc-54 3'UTR, myo-3p::mCherry]; rde-1(ne219) V; jyIs8 X*	1C-E, S4D	This study	
ERT1151	*mgTi54[rpl-28p::mScarlet::drh-1]; drh-1(jy110) IV; rde-1(ne219) V*	5C	(14)	
ERT1182	*jyEx305[vha-6p::drh-1-NTD::wrmScarlet::3xFLAG::unc-54 3'UTR]; rde-1(ne219) V; jyIs8 X*	1C/D, 2A-C, S2C	This study	
ERT1199	*jyEx305; rde-1(ne219) V*	1F, 3A/B, S4C	This study	
ERT1205	*jyEx305; zip-1(jy14) III; rde-1(ne219) V; jyIs8 X*	2A/B	This study	
ERT1207	*jyEx304[vha-6p::drh-1-NTD::wrmScarlet::3xFLAG::unc-54 3'UTR]; rde-1(ne219) V; jyIs8 X*	1C/D, S1A/B	This study	
ERT1208	*jyEx304; rde-1(ne219) V*	3A-E, S4A, S7A-C	This study	
ERT1209	*jyEx302; rde-1(ne219) V*	3A/B, S4E	This study	
ERT1215	*frSi17[mtl-2p::rde-1 3'UTR] II; rde-1(ne300) V; jyIs8 X*	4	(15)	IG1839 from Watts et al., 2020 was crossed with ERT054 in this study
ERT1216	*frSi21[col-62p::rde-1 3'UTR] II; rde-1(ne300) V; jyIs8 X*	4	(15)	IG1846 from Watts et al., 2020 was crossed with ERT054 in this study
ERT1257	*jyEx305; zip-1::gfp(jy132) III; rde-1(ne219) V*	S3	This study	
ERT1262	*jyEx336[vha-6p::wrmScarlet::3xFLAG 3'UTR]; rde-1(ne219) V; jyIs8 X*	S1C	This study	
ERT711	*rde-1(ne219) V; jyIs8 X*	1C/D, 2A/B, S1B	This study	
ERT781	*drh-1(jy110)*	S6	(16)	
GR3325	*mgTi54[rpl-28p::mScarlet::drh-1]*	S6	(14)	
N2	wild type	S6	Caenorhabditis Genetics Center	
WM27	*rde-1(ne219) V*	1F, S4B	Caenorhabditis Genetics Center	
ERT1306	*jyEx353; rde-1(ne219) V*	S7D-F	This study	
ERT1287	*jyEx353[vha-6p::drh-1-HC::wrmScarlet]; rde-1(ne219) V; jyIs8 X*	S1D	This study	
ERT1310	*jyEx304; jyIs14[F26F2.1p::GFP, myo-2::mCherry] V*	S2A/B	This study	

Table S2. Constructs used in this study.

Construct name	Description	Source
pET636	*vha-6p::pals-22 cDNA no stop::gfp_SBP_3xFLAG::unc-54 3'UTR* in pCFJ151	(17)
pET770	*vha-6p::drh-1(2CARD)::wrmScarlet::3xFLAG::unc-54 3'UTR, unc-119(+)*	This study
pET786	*vha-6p::wrmScarlet::3xFLAG::unc-54 3'UTR, unc-119(+)*	This study
pET788	*vha-6p::drh-1::wrmScarlet::3XFLAG::unc-54 3'UTR, unc-119(+)*	This study
pET794	*vha-6p::drh-1(HC)::wrmScarlet::3xFLAG::unc-54 3'UTR, unc-119(+)*	This study
Tian233	*rpl-28p::mScarlet::drh-1::unc-119(+), Mos1*	(14)

Table S3. Primers used in this study.

Primer name	Sequence	Purpose
LEB001	TACAAATCAGGATCAGGATCAGC	Cloning for intestinal expression of *drh-1(2CARD)*
LEB002	CCCGGGTTTATGGGTTTTGG	Cloning for intestinal expression of *drh-1(2CARD)*
LEB003	ATGGTCAGCAAGGGAGAGG	Cloning for empty vector control
LEB004	CCCGGGTTTATGGGTTTTGG	Cloning for empty vector control
LEB005	TTCAGGGCGACTAAAACCTACCAAAACCCAT AAACCCGGGAGGAAAAAGCAGTGTTCTTC	Cloning for intestinal expression of *drh-1*
LEB006	GAACTCCTTGATAACTGCCTCTCCCTTGCTG ACCATTGCTTCTCTGATTAAATTGACTAC	Cloning for intestinal expression of *drh-1*
LEB007	GTAATTGAATCTGTTCGTCAACGAATTC	Cloning for intestinal expression of *drh-1(HC)*
LEB008	CATCCCGGGTTTATGGGTTTTG	Cloning for intestinal expression of *drh-1(HC)*
snb-1 F	CCGGATAAGACCATCTTGACG	qPCR
snb-1 R	GACGACTTCATCAACCTGAGC	qPCR
pals-5 F	CATTGGAAAGCGATATTGGA	qPCR
pals-5 R	TCTCCAGGCACCTATCTTGTAG	qPCR
F26F2.1 F	TGGAACCAGGTCAGAGACAC	qPCR
F26F2.1 R	TTGTGAGAATTTCCGCGATA	qPCR
skr-5 F	CGAAGAGCAAGATGTCAAAATTG	qPCR
skr-5 R	AGAAGCTTGGATTGATTGGCA	qPCR
cul-6 F	CTGGGCTTACTCACAATGCC	qPCR
cul-6 R	GCAGAGTTGGCTTGCTGTAA	qPCR
eol-1 F	GAAGGAGGTGGCGATGTTTAT	qPCR
eol-1 R	CGGCGTCGATTGTCTCTTT	qPCR
RNA1 F	ACCTCACAACTGCCATCTACA	qPCR
RNA1 R	GACGCTTCCAAGATTGGTATTGGT	qPCR
C18D4.4 F2	GCAGGCATGTACTTTACTTGGGC	qPCR
C18D4.4 R2	TTTGCCACGCTTTCCATGTTCC	qPCR
B0507.5 F	AATGGAGAATCATGCCACCA	qPCR
B0507.5 R	ACTCCTCAATCGCTGCATCA	qPCR
T27E7.9 F	TTCGTGAGCATGAATACTGAAGA	qPCR
T27E7.9 R	CTGATTGGAACTAGGAAGAAGATTG	qPCR
math-38 F	ATGGGGGTCGCTGAAGAATG	qPCR
math-38 R	ACGGATTTGGAGCTTCCACC	qPCR
clec-60 F	TCCGTGCTCTTTCGTCAGTCAGC	qPCR
clec-60 R	TGTACCCTTGTTTGCCGGCTTCA	qPCR
drh-1 F	GTTCGAAAAACTCGCCTGA	qPCR
drh-1 R	TTTGTCAAGAATATTTTTCCATTTC	qPCR

Dataset S1 (separate file). Foldseek analysis

Dataset S2 (separate file). Dali analysis

SI References

1. J. Jumper, *et al.*, Highly accurate protein structure prediction with AlphaFold. *Nature* **596**, 583–589 (2021).

2. M. Varadi, *et al.*, AlphaFold Protein Structure Database: massively expanding the structural coverage of protein-sequence space with high-accuracy models. *Nucleic Acids Research* **50**, D439–D444 (2022).

3. M. van Kempen, *et al.*, Fast and accurate protein structure search with Foldseek. *Nat Biotechnol* 1–4 (2023). https://doi.org/10.1038/s41587-023-01773-0.

4. L. Holm, Dali server: structural unification of protein families. *Nucleic Acids Research* **50**, W210–W215 (2022).

5. L. Holm, A. Laiho, P. Törönen, M. Salgado, DALI shines a light on remote homologs: One hundred discoveries. *Protein Science* **32**, e4519 (2023).

6. J. Goudeau, *et al.*, Split-wrmScarlet and split-sfGFP: tools for faster, easier fluorescent labeling of endogenous proteins in Caenorhabditis elegans. *Genetics* **217** (2021).

7. M. W. Pfaffl, A new mathematical model for relative quantification in real-time RT-PCR. *Nucleic Acids Res* **29**, e45 (2001).

8. V. Lažetić, *et al.*, The transcription factor ZIP-1 promotes resistance to intracellular infection in Caenorhabditis elegans. *Nat Commun* **13**, 17 (2022).

9. M. A. Bakowski, *et al.*, Ubiquitin-Mediated Response to Microsporidia and Virus Infection in C. elegans. *PLOS Pathogens* **10**, e1004200 (2014).

10. K. M. Balla, E. C. Andersen, L. Kruglyak, E. R. Troemel, A wild C. elegans strain has enhanced epithelial immunity to a natural microsporidian parasite. *PLoS Pathog* **11**, e1004583 (2015).

11. J. Panek, *et al.*, A cullin-RING ubiquitin ligase promotes thermotolerance as part of the intracellular pathogen response in Caenorhabditis elegans. *PNAS* **117**, 7950–7960 (2020).

12. P. Bankhead, *et al.*, QuPath: Open source software for digital pathology image analysis. *Sci Rep* **7**, 16878 (2017).

13. K. Chen, C. J. Franz, H. Jiang, Y. Jiang, D. Wang, An evolutionarily conserved transcriptional response to viral infection in Caenorhabditis nematodes. *BMC Genomics* **18**, 303 (2017).

14. K. Mao, P. Breen, G. Ruvkun, Mitochondrial dysfunction induces RNA interference in C. elegans through a pathway homologous to the mammalian RIG-I antiviral response. *PLoS Biol* **18**, e3000996 (2020).

15. J. S. Watts, *et al.*, New Strains for Tissue-Specific RNAi Studies in Caenorhabditis elegans. *G3 (Bethesda)* **10**, 4167–4176 (2020).

16. J. N. Sowa, *et al.*, The Caenorhabditis elegans RIG-I Homolog DRH-1 Mediates the Intracellular Pathogen Response upon Viral Infection. *J Virol* **94**, e01173-19 (2020).

17. K. C. Reddy, *et al.*, An Intracellular Pathogen Response Pathway Promotes Proteostasis in C. elegans. *Curr Biol* **27**, 3544-3553.e5 (2017).
